# Unveiling the complexity of the maize transcriptome by single-molecule long-read sequencing

**DOI:** 10.1038/ncomms11708

**Published:** 2016-06-24

**Authors:** Bo Wang, Elizabeth Tseng, Michael Regulski, Tyson A Clark, Ting Hon, Yinping Jiao, Zhenyuan Lu, Andrew Olson, Joshua C. Stein, Doreen Ware

**Affiliations:** 1 Cold Spring Harbor Laboratory, One Bungtown Road, Cold Spring Harbor, New York 11724, USA; 2 Pacific Biosciences, 1380 Willow Road, Menlo Park, California 94025, USA; 3 USDA ARS NEA Robert W. Holley Center for Agriculture and Health Cornell University, Ithaca, New York 14853, USA

## Abstract

*Zea mays* is an important genetic model for elucidating transcriptional networks. Uncertainties about the complete structure of mRNA transcripts limit the progress of research in this system. Here, using single-molecule sequencing technology, we produce 111,151 transcripts from 6 tissues capturing ∼70% of the genes annotated in maize RefGen_v3 genome. A large proportion of transcripts (57%) represent novel, sometimes tissue-specific, isoforms of known genes and 3% correspond to novel gene loci. In other cases, the identified transcripts have improved existing gene models. Averaging across all six tissues, 90% of the splice junctions are supported by short reads from matched tissues. In addition, we identified a large number of novel long non-coding RNAs and fusion transcripts and found that DNA methylation plays an important role in generating various isoforms. Our results show that characterization of the maize B73 transcriptome is far from complete, and that maize gene expression is more complex than previously thought.

## Abstract (web summary)

*Zea mays* is an important crop species and genetic model but uncertainties remain regarding the structure of the transcriptome. Here Wang *et al*. use single-molecule sequencing and size-fractionated libraries to identify novel transcripts and isoforms illustrating the complexity of maize mRNA.

## Graphical abstract

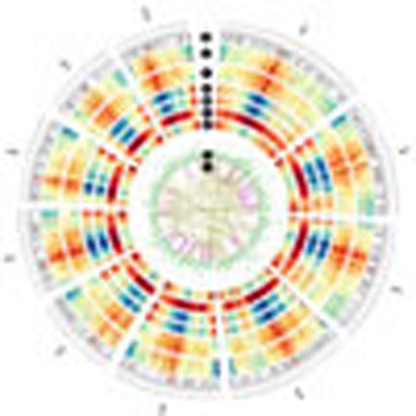

Maize (*Z. mays*), one of the world’s most important crops, is a leading model for elucidating plant transcriptional networks. Research in this species is facilitated by increasingly refined knowledge of the transcriptome across spatio-temporal, developmental and environmental dimensions^1^,^2^,^3^. The maize genome reference assembly, released in 2009, used a minimum bacterial artificial chromosome (BAC) tiling path strategy aimed at completely covering the gene space; the BACs used in this strategy were anchored by genetic and physical maps^4^,^5^. The reference assembly and gene space annotations were further refined, and the genes were annotated using both evidence-based approaches (for example, complementary DNA, expressed sequence tag (EST) and RNA-sequencing (RNA-seq) data) and an *ab initio* approach, which were combined to yield a non-redundant gene set^6^,^7^. Although data from short-read sequencing have accumulated over recent years, they do not provide full-length (FL) sequence for each RNA, limiting their utility for defining alternatively spliced forms; furthermore, in some cases, short-read sequencing generates low-quality transcripts, leading to incorrect annotations^8^.

Alternative splicing is prevalent in most eukaryotic genomes^9^,^10^,^11^. This phenomenon, which greatly increases the repertoire of proteins, regulates molecular, cellular, physiological and developmental processes/pathways^12^,^13^. In animals, expansion of transcript isoforms from multi-exon genes is thought to have facilitated the evolution of functional complexity without massively increasing the number of genes. In *Drosophila*, alternative splicing of the *DSCAM* gene generates over 38,000 isoforms, more than twice the number of genes in the genome^14^. In human^15^ and the model plant *Arabidopsis*^16^, respectively, ∼95% and 61% of multi-exonic genes are alternatively spliced. The functional significance of most multiple-transcript isoforms is poorly understood, but well-studied cases demonstrate that alternative splicing is a regulatory mechanism with profound consequences for organismal function. In *Drosophila*, the *Bcl-x* gene produces two transcript isoforms, one that inhibits apoptosis and another that activates it^17^. In addition, sex-specific alternative splicing in fly governs sex determination by functionally regulating several developmental genes^18^. In *Arabidopsis*, alternative splicing of zinc-induced facilitator-like 1 (*ZIFL1*) yields two isoforms: *ZIFL1.1* influences cellular auxin efflux and polar auxin transport in roots, whereas *ZIFL1.3* regulates stomatal movement^19^. Several modes of alternative splicing exist^20^: exon skipping predominates in animals, whereas intron retention (IR) predominates in plants^21^.

Established methods such as Sanger sequencing of FL cDNA clones are the most reliable means of transcript discovery and have historically represented the gold standard for genome annotation projects^22^. However, these methods fell out of fashion with the advent of cheaper short-read technologies. Short reads require computational assembly; therefore, although this approach accurately discovers splice sites, it is difficult to infer the actual combinations of splice-site usage, limiting the accuracy of gene model prediction. By producing longer reads, the PacBio single-molecule technology eliminates the need for assembly^8^,^23^,^24^,^25^, providing direct evidence for transcript isoforms of each gene. This methodology provides superior evidence for alternative splicing and can often improve the accuracy of existing gene models^8^,^24^,^25^.

We applied PacBio long-read technologies to transcriptome sequencing in maize. To ensure wide coverage of transcript isoforms, many of which may be tissue specific, we multiplexed six tissues at different developmental stages (root, pollen, embryo, endosperm, immature ear and immature tassel) and pooled them for size selection and subsequent sequencing. In parallel, messenger RNA from these tissues was sequenced on the Illumina HiSeq 2000 PE101 platform to quantify gene/isoform expression. The results constitute a rich data set of FL cDNA sequences that extends our knowledge of the maize transcriptome far beyond existing resources in terms of breadth (genes covered) and depth (transcripts per gene). Our results demonstrate the PacBio Iso-Seq platform’s reliability and its utility in characterizing FL cDNA transcripts and identifying novel genes/isoforms, which will improve genome annotation and enhance our understanding of the maize transcriptome.

## Results

### FL sequencing and bioinformatics pipeline

**Figure 1:**
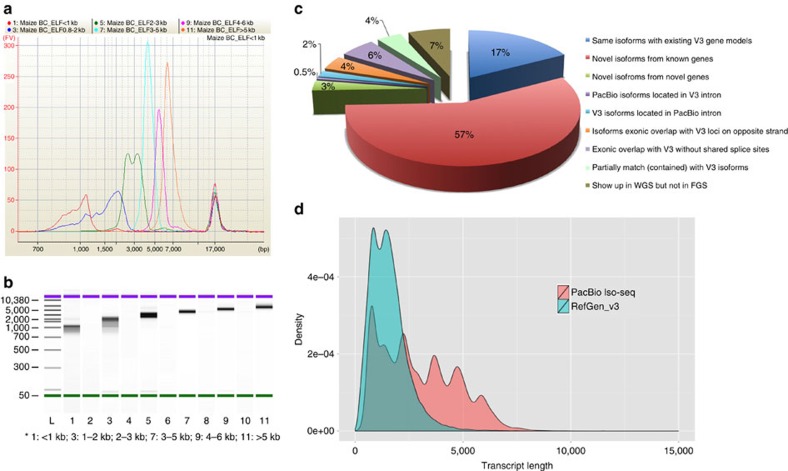
Maize PacBio Iso-Seq barcoding library and comparison of isoforms between RefGen_v3 and PacBio data. (**a**) Quantification of six size-fractionated libraries on a Bioanalyzer chip. (**b**) Gel image of six size-fractionated libraries on a Bioanalyzer chip. (**c**) Comparison of PacBio and RefGen_v3 isoforms. (**d**) Comparison of isoform length between RefGen_v3 and PacBio data.

To identify as many transcripts as possible, high-quality (HQ) RNA was extracted from six tissues of the maize inbred line B73 at different developmental stages and reverse transcribed (Supplementary Fig. 1). Tissue-specific barcodes were added before pooling for subsequent amplification. To avoid loading bias, which favours sequencing of shorter transcripts, multiple size-fractionated libraries (<1, 1–2, 2–3, 3–5, 4–6 and >5 kb) were made using a SageELF device (Fig 1a,b). Barcoded SMRTBell libraries were sequenced on the PacBio RS II using the latest P6–C4 chemistry with 47 SMRT cells, yielding 3,716,604 reads (Supplementary Table 1), and processed using ToFU^23^ (Supplementary Fig. 2a). Each size-selected library had the expected distribution of transcript lengths, ranging from 300 to 8,000 bp (Supplementary Fig. 2b). Almost half (1,553,692, 43%) of reads were classified as FL based on the presence of barcoded primers and polyA tails. ToFu processing yielded 643,330 FL, HQ consensus transcript sequences. HQ transcript sequences were mapped to the maize RefGen_v3 genome assembly using GMAP^26^, and 606,145 (94.2%) mapped sequences were collapsed into 111,151 non-redundant isoforms (see Methods). To minimize inclusion of possible truncated transcripts due to incomplete reverse transcription, reads differing only at the 5′-start site within the first exon were counted as redundant and only the longest version was retained. We discarded 37,185 (5.8%) sequences due to low coverage and identity.

### Isoform detection and characterization

Of the 111,151 unique isoforms, 829 were transposable elements (TEs) including 489 (59%) long terminal repeat (LTR)/Gypsy, 273 (33%) LTR/Copia, 29 (3%) terminal inverted repeats (TIRs), 15 (2%) long interspersed nuclear elements (LINEs) and 23 (3%) unknown LTRs. Among the 6 tissues, endosperm had the most TEs (333) and tassel had the fewest (Supplementary Fig. 3).

**Figure 2:**
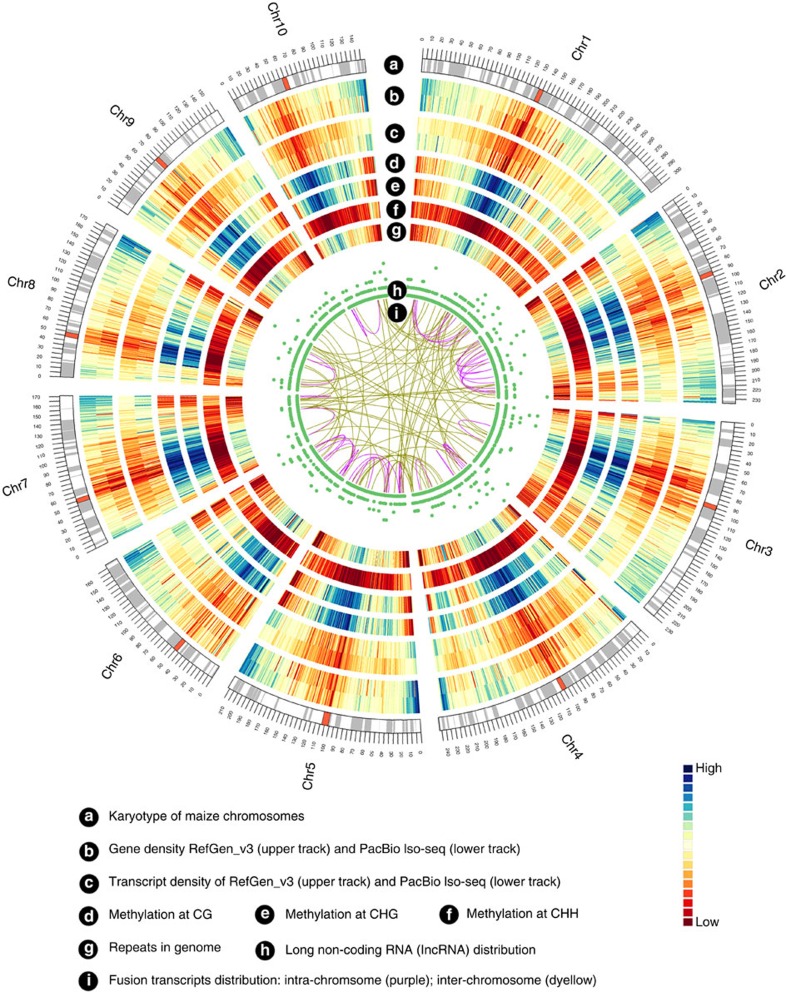
CIRCOS visualization of different data at the genome-wide level. (**a**) Karyotype of maize genome. (**b**) Comparison of gene density between genes covered by RefGen_v3 and the PacBio data set. Gene density was calculated in a 1-Mb sliding window at 20 kb intervals. (**c**) Comparison of isoform density between RefGen_v3 and PacBio sequences; isoforms density was calculated in a 1-Mb sliding window at 20 kb intervals. (**d**) CG methylation level. (**e**) CHG methylation level. (**f**) CHH methylation level. Each methylation in 1 Mb bins on each chromosome. (**g**) Repeat density in genome. (**h**) lncRNA density, in 1 Mb bins on each chromosome. (**i**) Linkage of fusion transcripts: purple, intra-chromosomal; dark yellow, inter-chromosomal.

We compared the remaining 110,322 isoforms against the maize RefGen_v3 annotation build 5b+ filtered gene set (FGS) and 5a working gene set (WGS). The FGS contains 63,540 transcripts covering 39,656 loci, whereas the WGS includes the FGS plus additional low-confidence annotations for a total of 136,770 transcripts covering 110,028 loci. Unique isoforms covered ∼70% of RefGen_v3 FGS loci; overall, gene density was lower than in 5b+, albeit higher in some specific regions (Fig. 2a,b). We classified isoforms into eight groups (Fig. 1c and Supplementary Fig. 4a–h) as follows: (a) 2,803 (3%) novel transcripts from 2,253 novel loci (that is, absent from both V3 FGS and WGS). To better understand the origin and coding potential of these loci, we aligned them to annotated proteins in the related species sorghum, rice and *Brachypodium* (BLASTX; e-value ≤1e−10). Around half (1,325, 47.3%) exhibited homology to at least one genome and 866 (30.9%) were present in all three genomes; the remaining isoforms (1,478, 52.7%) exhibited no homology to any annotated genes (Supplementary Fig. 5a), suggesting that they are species specific. (b) Novel isoforms (62,547, 57%) that share at least one splice site with the V3 annotated genes/isoforms, but differ at other splice sites. Among these, 62,053 (99.2%) were homologous to annotated proteins in other grasses (Supplementary Fig. 5b). (c) Isoforms (19,012, 17%) that share the same intron chain and splice sites with existing maize V3 gene models. (d) Isoforms (6,123, 5.6%) with exonic overlap with V3 gene models but without shared splice sites. (e) V3-annotated transcripts (2,014, 1.8%) located in the introns of PacBio isoforms; by contrast, 497 (0.5%) PacBio isoforms were located in the intron of a V3-annotated isoform. (f) Isoforms (4,368, 4%) with exonic overlap with a V3-annotated locus on the opposite strand. (g) Isoforms (4,304, 3.9%) partially matching V3 transcripts, that is, PacBio transcripts that share splice sites at matched regions but are shorter than annotated V3 sequence. (h) Transcripts (2,199, 2.9%) covering more than one annotated V3 gene, suggesting mis-annotation of single genes as multiple genes in V3. Such mis-annotation (a ‘split gene model’) is common in both plant and animal genome annotation projects. To evaluate agreement of our data with these predictions, reads were compared with the current list of 844 putative split gene models downloaded from Gramene^6^ (ftp://ftp.gramene.org/pub/gramene/CURRENT_RELEASE/data/split_genes/Split_genes_b46/Zea_mays.txt). Mis-annotation was confirmed in all cases for which a read was available, 682 (81%) in all. In 42 cases, the V3 model was partially correct, predicting at least one transcript isoform produced by the merged gene (Supplementary Fig. 6a,b).

By size selecting for longer transcripts, our single-molecule sequencing data recovered transcripts longer than those described in the current B73 annotations (Fig. 1d). We compared the FL PacBio cDNAs with a previously published FL cDNA project^27^,^28^,^29^; of 69,306 cDNA sequences downloaded from GenBank, 29,881 overlapped with 16,485 PacBio sequences. Moreover, the PacBio isoforms (median, 2,632 bp) were much longer than the GenBank FL isoforms (1,160 bp) (Supplementary Fig. 7a,b). In the current V3 maize annotation, 14,536 genes are annotated with two or more isoforms; the locus with the largest number of isoforms (15) is chr8:134,051,427–134,069,240 (gene ID: GRMZM2G067985). In the PacBio data set, we identified 15,146 genes with two or more isoforms and significantly more isoforms per gene (Supplementary Fig. 8a), with an average of 6.56 isoforms, more than twice the number in the V3 FGS annotation (2.84); the locus with the largest number of isoforms (324) is chr4:4,172,268–4,182,268 (gene ID: PB.12344). Thus, the PacBio data had higher isoform density than RefGen_v3 at the whole-genome level (Fig. 2c). In addition, the number of isoforms per gene positively correlated with the number of introns (Supplementary Fig. 8b), whereas the expression level did not significantly correlate with isoform number (Supplementary Fig. 8c).

### Read depth analysis

Rarefaction analysis revealed that sequencing depth, achieved using 47 multiplexed SMRT cells, had reached near-saturation of gene discovery within those size ranges (Supplementary Fig. 9). At this coverage level, 70% of RefGen_v3 genes were represented in our transcriptome sequences. Recent re-evaluation of RefGen_v3 using extensive expression data showed that >6% of RefGen_v3 genes have no supporting evidence and may represent false-positive predictions^30^. Therefore, the observed gene-discovery rate is likely to be an underestimate. To further investigate the relationship between sequencing depth and transcript discovery, we performed similar analyses using reads from individual tissues or different size-fractionated libraries pooled across tissues. Discovery of known RefGen_v3 transcript isoforms reached saturation much sooner (that is, at lower subsample sizes) in pollen and to a lessor extent in endosperm than reads from other tissues, especially root (Supplementary Fig. 10a). Saturation was also reached sooner in reads from larger-insert libraries, with a clear trend towards decreasing transcript diversity with increasing insert length, except that the 1–2 kb library was more diverse than the 0–1 kb library (Supplementary Fig. 10b). Similar results were obtained using novel and known transcript isoforms as outcome measures (Supplementary Fig. 10c,d).

### Tissue-specific isoforms and alternative splicing modes

**Figure 3:**
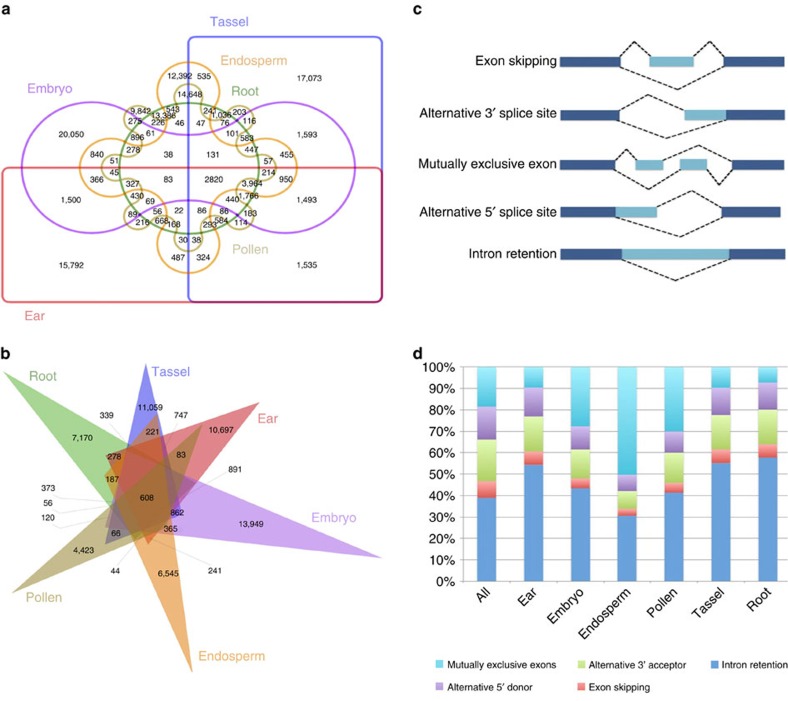
Comparison of different isoforms among six tissues and different alternative splicing modes. (**a**) Overlap of all PacBio isoforms in six tissues. (**b**) Overlap of novel isoforms among six tissues. (**c**) Visualization of five alternative splicing modes. (**d**) Distribution of different types of alternative splicing events in six tissues.

Of the 6 tissues, pollen had the highest proportion of tissue-specific isoforms (9,842; 61.3%), followed by embryo (20, 050; 49.2%) and endosperm (12, 392; 46.7%), whereas root had the lowest (13,386; 44.6%) (Fig. 3a). Comparison of novel tissue-specific isoforms generated from both known and novel genes revealed similar patterns (Fig. 3b). Gene ontology analysis showed that tissue-specific isoforms are enriched for particular molecular functions that vary with tissue. In endosperm, tissue-specific transcripts were enriched for nutrient reservoir function, consistent with the role of endosperm in food storage. By contrast, pollen-specific transcript isoforms were enriched in enzyme regulators (Supplementary Fig. 11a–f).

To ascertain the relative importance of the five main modes of alternative splicing (intron retention, exon skipping, alternative 3′-acceptor, alternative 5′-donor and mutually exclusive exon) in each tissue, we used AStalavista^31^ (Fig. 3c and Supplementary Data 1). IR predominated, accounting for 40% of alternative transcripts. To exclude the possibility that these transcripts were experimental or informatics artefacts, we performed independent validation by quantitative PCR. All ten randomly selected IR events were successfully validated (Supplementary Fig. 12), confirming the high fidelity of the long-read sequencing strategy. IR can introduce stop codons, thereby activating nonsense-mediated decay^32^, but can also change open reading frames (ORFs), leading to functionally different variants (Supplementary Fig. 13). Alternative 3′-splicing was the second most prevalent (19%) mode, whereas exon skipping (7.8%) was the least frequent.

Splicing mode was not uniform across the six tissues. In particular, mutually exclusive exon splicing, in which transcripts use either of two exons, predominated in endosperm and was more prevalent in pollen and embryo than in ear, tassel and root (Fig. 3d). Moreover, multiple splicing modes could operate on a single transcript, potentially combinatorially generating diverse isoforms from a single gene. For example, various combinations of alternative 5′-donors and 3′-acceptors, along with exon skipping and IR, yielded 42 observed isoforms of the PacBio gene PB.24528 (GRMZM5G878615) (Supplementary Fig. 14). Some of these isoforms were tissue specific due to differential use of these modes.

### Transcription factor isoforms produce functional variants

The maize V3 annotation contains 2,624 annotated transcription factors (TFs) from 57 families. Using our data, we identified novel isoforms for 53 families, increasing the number of TF isoforms to 5,423. Although this increase was broadly distributed (41 of the 57 TF families; Supplementary Fig. 15a), some families had a particularly high prevalence of isoforms. For example, the V3 annotation contains 38 members of the auxin response factor family; our data revealed 155 new isoforms, nearly tripling the number of annotated variants. As auxin response factor TFs are important regulators of auxin transport, we believe these novel isoforms will provide additional mechanistic insights into the role of auxin metabolism in plant development. Additional noteworthy families with expanded numbers of transcript isoforms include Homeobox, FAR1-like and MADS-box family (Supplementary Fig. 15a). Expression analysis revealed that many TF families were more highly expressed in pollen (Supplementary Fig. 15b) and the 5,423 TF isoforms were classified into 20 clusters by Mfuzz^33^ based on their expression value (Supplementary Fig. 16).

### LncRNA identification

**Figure 4:**
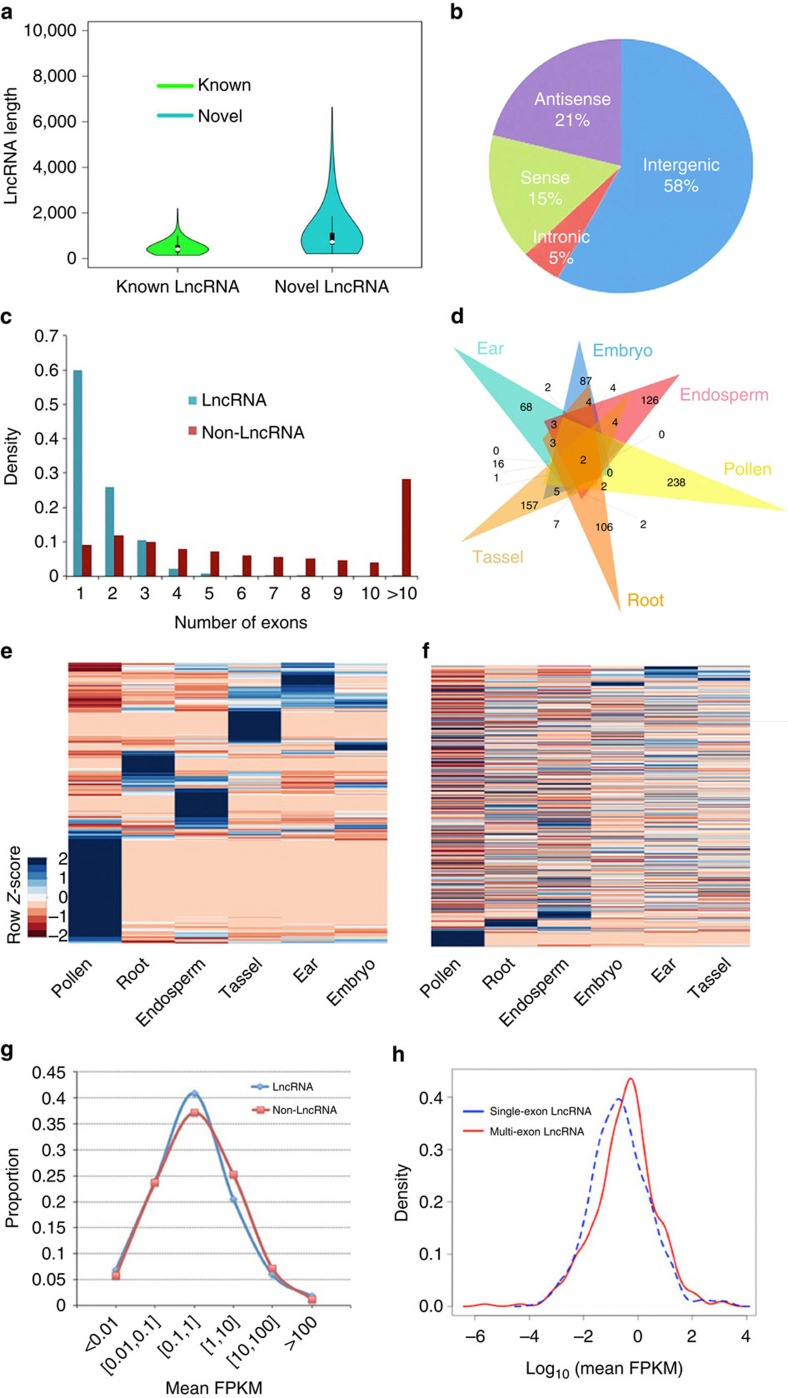
Characters of identified novel lncRNAs. (**a**) Comparison of lengths of novel lncRNAs identified in this study with previously reported lncRNAs. (**b**) Proportions of four kinds of lncRNA, classified according to biogenesis. (**c**) Number of exons of lncRNAs and non-lncRNAs. (**d**) Overlap of lncRNAs among six tissues. (**e**) Heatmap of lncRNA expression in six tissues. (**f**) Heatmap of non-lncRNA expression in six tissues. (**g**) Comparison of overall expression between lncRNAs and non-lncRNAs. (**h**) Comparison of overall expression between single-exon lncRNAs and multi-exon lncRNAs.

In addition to protein-coding RNAs, non-coding RNAs constitute a major component of the transcriptome. Recently, 1,704 high-confidence long non-coding RNAs (lncRNAs) (mean length, 463 bp) were identified in maize by Illumina short-read sequencing^34^. To identify lncRNAs in the PacBio data, we built a classification model using PLEK^35^, trained on a high-confidence set of known non-coding RNA genes^34^,^36^. Scanning of single-molecule long reads revealed 12,226 candidate lncRNAs of ⩾200 bp. To obtain a high-confidence set of lncRNA genes, we eliminated transcripts with ORFs exceeding 100 codons and used BLASTX to screen the remaining 1,290 candidates for homology with proteins of sorghum, rice and *Brachypodium*, thereby eliminating 412 genes (Supplementary Fig. 17). BLASTN revealed that 11 of the remaining 878 candidates corresponded to previously discovered lncRNAs. The remaining 867 novel high-confidence lncRNAs had a mean length of 1.1 kb (range, 0.2–6.6 kb) (Fig. 4a). We classified them into four groups based on their positions relative to RefGen_v3 annotations: 58% of them were generated from intergenic regions, 21% from the antisense strand, 16% from the sense strand and 5% from intronic regions (Fig. 4b). In addition, 519 (59.9%) of the lncRNAs were single exon (Fig. 4c). Pollen expressed the most specific lncRNAs (238) and immature ear expressed the fewest (68) (Fig. 4d). Expression profiling confirmed that lncRNAs exhibit tissue-specific expression (Fig. 4e,f) and are usually less highly expressed than non-lncRNAs (Fig. 4g), although multi-exon lncRNAs were expressed at higher levels than single-exon lncRNAs (Fig. 4h). Mapping lncRNAs to chromosomes revealed that they have similar distribution to that of protein-coding genes, which are enriched outside of pericentromeric regions, and associated with low-repeat and low-CG/CHG methylation regions (Fig. 2d–h).

### Fusion transcript identification

In this study, we identified 1,430 fusion transcripts (see Methods). Validation using an Illumina pair-end read approach revealed that 134 fusions were supported by at least one uniquely mapping paired-end read (Fig. 2I and Supplementary Data 2). An important observation is that the site of transcript fusion corresponded with splice junctions that also function within non-fused versions of these transcripts. This indicates that generation of fusion transcripts involves the splicing machinery, suggesting either *trans*-splicing of distinct genes or splicing of chimeric genes formed by somatic chromosomal rearrangements. The largest number of fusion candidates was found in endosperm (50) and embryo (28), whereas tassel had the fewest candidates (15). Fusion events were more likely to occur inter-chromosomally (88) than intra-chromosomally (46) (Fig. 2i) and tended to occur near chromosome termini. To further validate the fusion transcripts, we randomly chose seven candidates for reverse transcription (RT) PCR and Sanger sequencing. Five (71%) of the seven fusions were experimentally validated, for example, the fusion gene PBfusion.1622 and PBfusion.1237 (Supplementary Fig. 18a), confirming the authenticity of these chimeric RNAs. Gene ontology analysis of fusion transcripts revealed that most were associated with nutrient reservoir activity in molecular function category and metabolic/cellular process in biological process category (Supplementary Fig. 18b).

### Verification and quantification of PacBio isoforms

**Figure 5:**
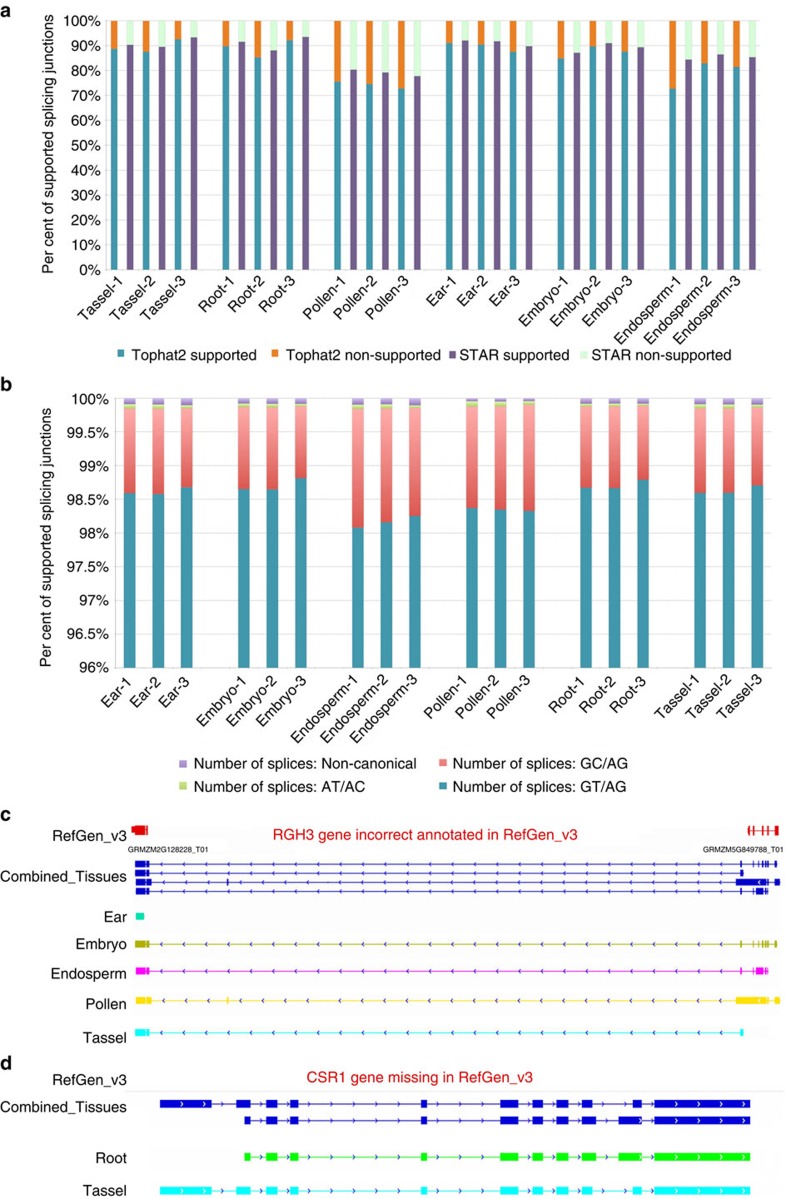
Validation of PacBio isoforms. (**a**) Verification of PacBio isoform junctions by short-read junction using TopHat2 and STAR. (**b**) Splicing motif distribution of short-read mapping by STAR. (**c**) The previously corrected *RGH3* gene model was found in the Iso-Seq data. (**d**) The previously missed *CSR1* gene model was found in the Iso-Seq data.

To verify and quantify the PacBio transcript isoforms, RNAs from three biological replicates of the same tissues used for PacBio sequencing were also sequenced on the Illumina HiSeq 2000 PE101 platform. Biological replicates correlated very well: Spearman’s correlation was >0.9 for all tissues (Supplementary Fig. 19). Short reads were mapped to the maize RefGen_v3 genome assembly using TopHat2.0.8b (ref. 37) and were compared with the PacBio genomic alignments. An average of 86% of splice junctions from six tissues in the PacBio data were supported by short-read mappings (Fig. 5a and Supplementary Fig. 20). As TopHat2 cannot detect all splicing motifs, we mapped short reads with another aligner, STAR^38^, which captures additional splice motifs including CT/AC, CT/GC, GT/AT and other non-canonical sites. STAR alignments provided more evidence supporting PacBio isoforms than TopHat2 alignments, increasing junction support to 90% (Fig. 5a). Relative to other tissues, pollen and endosperm had fewer short reads that supported PacBio-identified isoforms. Using the STAR results, we next compared splice motif preference among tissues and found that GC/AG motif was more prevalent in endosperm and pollen than in other tissues (Fig. 5b). In addition, we investigated incorrect gene models subsequently corrected by mapping clones and other experimental approaches. For example, *RGH3* (chr5:173,740,759–173,806,170) was reported to be a mis-annotation from the existing RefGen_v3 due to incorrect genome assembly^39^. In the PacBio data, we found four isoforms of *RGH3* (Fig. 5c): one shared a similar structure with the reported annotation and the other three were novel. Another example was *CSR1* on chr3:147,275,150–147,278,871; this locus has no gene model annotated in RefGen_v3 but recent work^40^ revealed that it indeed contains a gene. Our PacBio Iso-Seq data revealed two *CSR1* isoforms: one from root and the other from tassel (Fig. 5d).

To investigate the dynamics of novel isoforms over different stages of maize tissue development, uniquely mapped Illumina short reads from STAR were used to estimate normalized transcription level as fragments per kilobase of transcript per million mapped reads (FPKM) using Cufflinks^41^. To reduce transcription noise, we analysed only isoforms/genes with FPKM ⩾0.01, a value chosen based on gene coverage saturation analysis (Supplementary Fig. 21a). To facilitate graphical interpretation of relatedness among different tissues, we compared our results with four other available tissue-specific RNA-seq data sets^2^,^42^ from NCBI’s Sequence Read Archive, which investigated tissues of leaf, shoot, shoot apical meristem and seedling. Hierarchical clustering of the expression profiles revealed that pollen was most dissimilar to other tissues, followed by endosperm (Supplementary Fig. 21b), consistent with the fact that mature pollen has a highly specialized function in fertilization and contains only three cells, resulting in a distinctive expression profile.

### Comparison of PacBio isoforms with short-read assembly

The tremendous amount of alternative splicing of maize transcriptome identified by single-molecule long-read sequencing provides a good opportunity to assess the quality of short-read reconstruction. Two short-read assemblers were used to represent both reference-guided (Cufflinks^41^) and *de novo* (Trinity^43^) reconstruction strategies. To ensure a fair comparison, we only considered loci that were detected by both short reads and PacBio transcripts, and evaluated the assembled transcripts based only on their splicing junctions. Consequently, each assembler could only reconstruct a small percentage (Cufflinks: 22%; Trinity: 8%) of PacBio isoforms (Supplementary Fig. 22a). The performance of each assembler was further evaluated by its ability to recover PacBio isoforms. The results showed that the sensitivity of the two assemblers dropped sharply as isoform complexity increased (Supplementary Fig. 22b), indicating the limitations of short-read assembly methods for isoform discovery and suggesting that single-molecule long-read sequencing is essential for accurate isoform resolution, especially for genes with many isoforms.

### Methylation of isoforms

**Figure 6:**
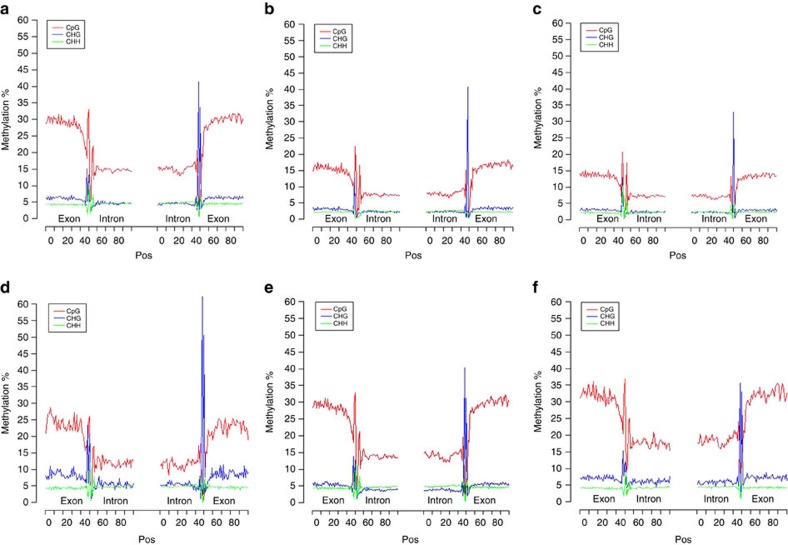
Level of DNA methylation at splice sites. (**a**) Level of DNA methylation, combing sense and antisense strand. (**b**) Level of DNA methylation on the sense strand. (**c**) Level of DNA methylation on the antisense strand. (**d**) Level of DNA methylation in genes with only one isoform. (**e**) Level of DNA methylation in all isoforms of genes with two to ten isoforms. (**f**) Level of DNA methylation in all isoforms of genes with more than 20 isoforms.

A previous methylation study based on a limited number of spliced genes in RefGen_v3 suggested that splice-site methylation inhibits alternative splicing^44^. The large number of isoforms identified by long-read sequencing in this work provided an opportunity to investigate the relationship between methylation and alternative splicing. To determine whether methylation level is related to generation of isoforms, we investigated DNA methylation levels of various isoforms. First, to determine the influence of DNA methylation on splicing, we stacked all splice junctions from the PacBio isoforms (see Methods) and measured the levels of three types of cytosine methylation (CpG, CHG and CHH, where H is A, C, or T) on both strands in each methylation context at donor and acceptor sites. The results revealed that CHG methylation was enriched at acceptor sites, whereas CG methylation was elevated at donor sites (Fig. 6a,b,c). To determine whether the methylation level was related to the number of isoforms, we separated the isoforms into three groups as follows: (a) genes with only 1 isoform (Fig. 6d); (b) genes with 2–10 isoforms (Fig. 6e); and (c) genes with ⩾20 isoforms (Fig. 6f). Using these groups, we investigated the DNA methylation level on all isoforms. Splicing was inversely correlated with CHG methylation at the acceptor site, suggesting that a higher level of CHG methylation represses alternative splicing and both the sense and antisense strands exhibited consistent methylation levels (Supplementary Fig. 23a–f). By contrast, CG methylation level at donor sites was positively correlated with the number of isoforms, suggesting that CG methylation promotes alternative splicing. Splicing and CHH methylation were not significantly correlated, although donor sites had higher CHH methylation levels than acceptor sites.

Next, we monitored levels of the three kinds of DNA methylation within and surrounding (1 kb upstream and 1 kb downstream) lncRNA and non-lncRNA isoforms. DNA methylation was reduced near the transcription start and stop sites for both lncRNA and non-lncRNA genes; non-lncRNA genes exhibited higher CG methylation within the gene body, whereas lncRNAs showed higher levels of CHG methylation. As gene body methylation is usually associated with gene expression^45^, this observation may reflect the differing expression levels of lncRNAs and non-lncRNA genes (Supplementary Fig. 24).

## Discussion

Large-scale sequencing of cDNA is instrumental for gene discovery and essential for genome annotation, but limitations in sequence technologies frequently force trade-offs between sampling depth and completeness. Although EST sequences constitute the majority of the current maize gene annotation, the sequences rarely cover the entire transcripts. By contrast, assembled FL cDNAs are the gold standard for annotation, but can be obtained for relatively small numbers of genes and at considerable cost. More recently, low-cost next-generation sequencing has provided deep insights into gene structure^30^, splice-site usage^46^, transcriptional networks^46^ and the maize pan-genome^3^. However, short reads prohibit accurate transcript reconstruction. Ultimately, long-read technologies are required to address the multiple requirements of scale, sampling depth, transcript completeness and cost.

Our experimental design was intended to maximize transcript diversity by broadly sampling six vegetative and reproductive tissues. For each tissue, we sampled six transcript size ranges (ranging from <1 to >5 kb) to counter both amplification and loading bias. PacBio sequencing yielded 111,151 unique FL transcript isoforms, corresponding to ∼26,946 genes. Rarefaction analysis revealed that final read depths had achieved near-saturation at the gene level. At the isoform level, saturation was attained in endosperm and pollen well below the final read depths, reflecting the lower transcriptome diversity of these tissues. Other tissues, in particular root, exhibited higher transcript diversity, raising the possibility that additional low-abundance transcript isoforms remain to be discovered. We chose not to include leaf tissue in this study, because the overabundance of transcripts involved in photosynthesis, such as the Rubisco small subunit, would necessitate far higher read depths to adequately sample transcript diversity.

A more important question is to what extent our sequencing strategy complements existing resources and provides advantages for discovery of novel or previously unrecognized protein-coding genes and transcript isoforms. Our analysis indicates that the new transcriptome data have enormous potential to improve the current maize annotation. The 111,151 unique transcripts characterized here almost double the number of transcripts documented in the RefGen_v3 annotation. Although most PacBio transcripts are novel isoforms of known genes (57%), 3% of these transcripts provide evidence for 2,253 novel genes. Moreover, 5.8% of the transcripts could not be mapped to the maize reference genome and thus represent genes that fall into gaps in the assembly or represent biological contaminants from endophytes or other sources.

Our analysis shows that long-read transcriptome data can correct a substantial number of mis-annotated gene models. As proof-of-concept, we examined two maize genes whose structures are well-characterized in the scientific literature but are improperly annotated in RefGen_v3. The *RGH3* locus was mis-annotated due to assembly error, whereas the *CSR1* locus, although present in the reference genome, lacks annotation altogether. Examination of the PacBio data yielded FL support for genes that match the known structures of the *RGH3* and *CSR1* genes, and also revealed alternative transcripts that further enrich our knowledge of these genes. Beyond these two examples, we expect that the new transcriptome data will greatly improve the gene annotation. As an illustration, we showed that FL transcripts could correct 682 out of 844 gene models predicted by comparative genomics to be falsely split.

Our strategy of using size-selected libraries of up to 10 kb enabled discovery of many transcripts that were much longer than those previously described. Indeed, the average length of transcripts captured in this study, nearly 3 kb, far exceeds the average length of the FL-cDNA collection and the average lengths of transcripts described in the RefGen_v3 annotation. These findings show that the prevalence of long transcripts, from both coding and non-coding genes, is higher than previously thought. Just as the availability of short-read technologies over the last decade heralded an era of tremendous gains in small RNA research, it is reasonable to expect that long-read technologies will prompt a new focus on heretofore poorly understood characteristics of exceptionally long RNAs.

LncRNAs, currently an emerging field in genome biology, are another area of particular neglect. Evidence from both plants and animals indicates that lncRNAs are rapidly evolving and are often species specific. What little is known about their function, mostly derived from studies in human cells, has revealed the roles of these RNAs in both transcriptional and epigenetic regulation. In this study, we identified an additional 867 high-confidence novel lncRNAs as single-molecule transcripts. Unexpectedly, the newly identified transcripts were exceptionally long, with a median length more than twice that of previously described lncRNAs. Consistent with previous work, we observed a high degree of tissue specificity among lncRNAs, a feature shared by other plant species^47^,^48^. Profiles of cytosine methylation within and around lncRNA genes were also similar to those previously described^34^,^45^. Our work solidifies the conclusion that lncRNAs and protein-coding genes share the predominant feature of reduced methylation at the start and end of the transcriptional unit, whereas differences in gene body methylation in these two types of genes may reflect quantitative differences in expression levels. Identification of these novel lncRNAs, many of which are intergenic, and some of which are as long as 6 kb, contributes substantially to our knowledge base and will enable future research into the functions of this intriguing class of RNA.

Our data set represents the single largest collection of FL cDNAs available in maize, including a rich diversity of isoforms ideal for studying alternative splicing. To further increase the reliability of this resource, we validated an average of 90% of splice sites from six tissues using high-depth Illumina reads. Our results agree with previous findings, including the estimated frequency of exon-skipping events and the predominance of IR as a mode of alternative splicing. However, we also made novel findings with respect to tissue specificity. In particular, in endosperm and pollen, we identified numerous cases of transcripts bearing alternative and mutually exclusive exons. These alternative isoforms may have functions related to the specialized characteristics of these tissues, for example, polyploidy in endosperm and microgametogenesis in pollen. As a precedent for this idea, maize endosperm expresses a tissue-specific non-functional isoform of cyclin that probably plays a role in endoreduplication, the developmental phase of endosperm, during which nuclear division continues in the absence of cytokinesis^49^. Molecular genetic analysis of maize *RGH3* gene also provided evidence that alternative splicing is required for cellular differentiation in maize endosperm^39^. Important questions remain regarding how alternative splicing is regulated. Notably in this regard, we found evidence that alternative splicing is repressed by CHG methylation at acceptor sites but promoted by CG methylation at donor sites.

Over the course of this study, we found many examples of chimeric transcripts that combine regions of different genes. Having considered possible mechanisms that may cause the formation of chimeric transcripts, including cloning artefacts and rare somatic chromosomal rearrangements, we found that these transcripts were mainly formed by a *trans*-splicing mechanism, carried out by joining exons from separate precursor transcripts. The key indication of *trans*-splicing was the observation that fusion boundaries between different RNAs corresponded to canonical splice sites that were also used by the respective transcripts during normal *cis*-splicing. To exclude the possibility that these chimeras were formed stochastically, we independently confirmed 134 of the splicing events using Illumina sequences. Thus, transcript fusion appears to be more common in maize than previously thought^50^, and chimeric fusion events further expand the complexity of the maize transcriptome. We also identified other characteristics of chimeric transcripts in maize, including the higher proportion of inter-chromosomal to intra-chromosomal fusions, and the higher prevalence of such transcripts in endosperm and embryos relative to other tissues.

Taken together, our study greatly improves existing gene models in maize and demonstrates that long-read sequencing complements short-read sequencing for cataloging and quantification of transcripts. As we continue to improve the maize genome assembly, we will update the maize genome annotation by adding novel isoforms, genes and lncRNAs, as well as gene/isoform profiling.

## Methods

### Plant materials

Maize inbred line B73 was grown at CSHL Uplands Farm. Roots were collected from 14-day-old seedlings; ears were collected from stage v8, tassels from stage v7, pollen from stage r1, and embryo and endosperm from seeds 20 days after pollination. Tissues were immediately frozen in liquid N_2_. For each tissue, at least 10 plants were pooled and ∼30 ears and tassels were pooled for each of three biological replicates.

### RNA preparation

Total RNA was prepared by grinding tissue in TRIzol reagent (Invitrogen 15596026) on dry ice and processed following the protocol provided by the manufacturer. To remove DNA, an aliquot of total RNA was treated with RQ1 DNase (Promega M6101), followed by phenol/chloroform/isoamyl alcohol extraction, chloroform/isoamyl alcohol extraction using Phase Lock Gel Light tubes (5 PRIME 2302800) and ethanol precipitation. Precipitated RNA was stored at −20 °C.

### Illumina RNA-Seq library construction

Total RNA (20 μg) was used for poly(A)^+^ selection using oligo(dT) magnetic beads (Invitrogen 610-02), eluted in water and subjected to RNA-seq library construction using the ScriptSeq kit (Epicentre SS10906). Libraries were amplified by 12–15 cycles of PCR and then sequenced in two lanes on the HiSeq 2000 PE101 platform at Woodbury Genome Center, Cold Spring Harbor Laboratory.

### Barcoding library and single-molecule sequencing

One microgram of total RNA per reaction per tissue was reverse transcribed using the Clontech SMARTer cDNA synthesis kit and tissue-specific barcoded oligo dT (with PacBio 16-mer barcode sequences; Supplementary Table 2) in separate PCR tubes, to generate barcoded FL cDNA. Two or three RT reactions per tissue were run in parallel. PCR optimization was used to determine the optimal amplification cycle number for the downstream large-scale PCR reactions. A single primer (primer IIA from the Clontech SMARTer kit 5′- AAG CAG TGG TAT CAA CGC AGA GTA C -3′) was used for all PCR reactions following RT.

Large-scale PCR products were purified with AMPure PB beads and quality control (QC) was performed on a 2100 BioAnalyzer (Agilent). Equimolar ratios of six cDNA libraries were pooled together. A total of 3.8 μg cDNA was subjected to size fractionation using the SageELF system. Size fractions eluted from the run were subjected QC and pooled in equimolar ratios for subsequent re-amplification to yield six libraries (<1, 1–2, 2–3, 3–5, 4–6 and >5 kb). The pooled PCR products were purified using AMPure PB beads.

One to five micrograms of purified amplicons were subjected to Iso-Seq SMRTBell library preparation (https://pacbio.secure.force.com/SamplePrep) Three barcoded SMRTBell libraries (3–5, 4–6 and >5 kb) were size-selected using the Sage BluePippin system to remove trace amounts of small inserts. A total of 47 SMRT cells (Supplementary Table 1) were sequenced on the PacBio RS II platform using P6-C4 chemistry with 3–4 h movies.

### Quantitative PCR validation

We randomly selected ten IR events from different PacBio isoforms for experimental validation. Specific primers of retained intron sequences (Supplementary Table 3) were used for SYBR Green-based quantitative real-time PCR analysis and *Zm18srRNA* was amplified as an endogenous control. Quantification results are expressed in terms of the cycle threshold (*C*_T_) value determined according to the manually adjusted baseline. Relative gene expression in different samples was determined using a previously described method^51^. Briefly, differences between the *C*_T_ values of target gene and endogenous control were calculated as Δ*C*_T_=*C*_T_target−*C*_T_endogenous control and expression levels of target genes relative to endogenous control were determined as 2^−Δ*C*_T_^. For each sample, PCR was repeated three times and the average values of 2^−Δ*C*_T_^ were used to determine the difference in gene expression. For validation of fusion transcripts, we randomly selected seven transcripts, designed specific primers for each sequence (Supplementary Table 4) and subjected the PCR products to Sanger sequencing.

### Illumina data analysis

The raw reads were aligned to the B73 reference genome (RefGen_v3) using TopHat2.0.8 and STAR with minimum intron length set to 20 bp and maximum intron length set to 50 kb, with default settings for other parameters. Quantification of genes and isoforms was performed using cufflinks version 2.2.1 using the GTF annotation file generated by PacBio sequencing. To reduce transcription noise, each isoform/gene was included for analysis only if its FPKM values was ⩾0.01, a value chosen based on gene coverage saturation analysis. A *k*-means approach was used (R Bioconductor package ‘Mfuzz’) to cluster 5,423 dynamically expressed TFs based on their expression profiles across different tissues. For short-read assembly, short-read transcripts were reconstructed by Cufflinks^41^ and Trinity^43^, assembled transcripts were mapped to the reference genome using GMAP^26^ and filtered for >85% alignment coverage and >90% alignment identity.

### Mapping of PacBio data

The PacBio raw reads were classified into Circular Consensus Sequences (CCS) and non-CCS subreads by ToFu^23^, by searching for the presence of sequencing adapters. Next, ToFU determines a CCS or subread sequence to be FL if both the 5′- and 3′-cDNA primers were present and there was a polyA tail signal preceding the 3′-primer. To improve consensus accuracy, we used an isoform-level clustering algorithm ICE (Iterative Clustering for Error Correction) and polished FL consensus sequences from ICE using Quiver. This method generated FL transcripts with ⩾99% post-correction accuracy. Mapping of FL CCS reads was carried out using GMAP^26^. Mapped FL reads were further aligned by BLASTN to all NCBI RefSeq, to consolidate the confidence, and collapsed by the pbtranscript-ToFU package (http://github.com/PacificBiosciences/cDNA_primer/) with min-coverage=85% and min-identity=90%, to collapse redundant transcripts, whereas the 5′-difference was not considered when collapsing the reads. Collapsed transcripts were screened for TEs by BLASTN alignment to a database of maize TE sequences (http://maizetedb.org/~maize/) with e-value ≤1e−10, min-coverage=85% and min-identity=90%. Each isoform was compared with existing gene models of RefGen_v3 annotation 5b+ by cuffcompare^41^ and the isoforms were further classified into eight groups based on their exon structures (splicing junctions). To examine overlap with existing cDNA resources available for maize, we retrieved 69,306 ‘FL_CDNA’ nucleotide sequences from NCBI and ran BLASTN with the following cutoff criteria: e-value ≤1e−10, min-coverage ⩾85% and min-identity ⩾90%.

### lncRNA identification from PacBio sequences

The 1,704 known high-confidence lncRNAs and microRNA precursors downloaded from Gramene 46 release were used to build a model using PLEK^35^. All PacBio isoforms were predicted based on the model and ORFs of candidate lncRNAs were predicted by EMBOSS. Transcripts encoding ORFs longer than 100 amino acids were filtered and the remaining transcripts were further screened by BLASTX (e-value ≤1e−10) against protein sequences of rice, *Brachypodium* and sorghum, downloaded from Gramene release b46 (http://ensembl.gramene.org/info/website/ftp/index.html). At a last step, BLASTN was used to get rid of the previously discovered lncRNAs under a criteria of e-value ≤1e−10, min-identity=90% and min-coverage=85%.

### Fusion transcript identification from PacBio sequences

To identify fusion transcripts, the criteria used for a single transcript were as follows: (a) FL transcripts map to two or more loci in the genome; (b) each mapped locus must align with at least 10% of the transcript; (c) the combined alignment coverage must be at least 99%; and (d) each mapped locus must be at least 100 kb apart. Furthermore, Illumina short reads generated from HiSeq2000 PE101 platform were used to validate candidate fusion transcripts, after mapping by STAR, junction spanning reads and discordant reads pairs were found to support the candidate fusion transcripts.

### Functional annotation of PacBio isoforms

To investigate the functions of all the isoforms, InterProScan-5.16-55.0 (ref. 52) (using default settings) was run to map known protein domains to all isoforms. Using InterPro to gene ontology (GO) mapping, GO terms were obtained for the InterPro domains. WEGO^53^ was used for the GO enrichment analysis comparing tissue specific isoforms to all isoforms.

### Methylation data analysis

Methylation data from Regulski *et al*.^44^ were remapped to the maize RefGen_v3 genome assembly and the methylation levels were calculated as previously reported. Briefly, all splice junctions from PacBio Iso-Seq transcripts were stacked (50 bp exon+50 bp intron for donor and 50 bp intron+50 bp exon for acceptor) and the methylation level of each base pair was calculated as C/(C+T). The methylation of the donor site was calculated from the first nucleotide of both strands on 5′-end of the intron as C/(C+T) and methylation of the acceptor site was calculated from the last nucleotide of both strands on the 3′-end of the intron using the same formula. For lncRNA and non-lncRNA methylation, three regions were used for methylation study: 1 kb upstream transcription start site (TSS), transcript body and 1 kb downstream transcription termination site (TTS). Each region was divided into 100 bins and the methylation ratio of each was calculated from the corresponding bins from all genes.

## Additional information

**Accession codes:** The PacBio data sets generated for this work is accessible through NCBI Sequence Read Archive under accession number SRP067440. The Illumina data is accessible through ArrayExpress accession number E-MTAB-3826.

**How to cite this article:** Wang, B. *et al*. Unveiling the complexity of the maize transcriptome by single-molecule long-read sequencing. *Nat. Commun.* 7:11708 doi: 10.1038/ncomms11708 (2016).

## Supplementary Material

Supplementary Information Supplementary Figures 1-24 and Supplementary Tables 1-4

Supplementary Data Set 1 Alternative splicing events of isoforms identified by PacBio Iso-Seq.

Supplementary Data Set 2 Characteristic of 134 fusion transcripts.
