# Supplementary material for: Unveiling the complexity of the maize transcriptome by single-molecule long-read sequencing
Source: Nat Commun. 2016 Jun 24;7:11708. doi: 10.1038/ncomms11708 (PMC4931018; doi:10.1038/ncomms11708)
Supplement: Supplementary Information — Supplementary Figures 1-24 and Supplementary Tables 1-4 [file ncomms11708-s1.pdf]

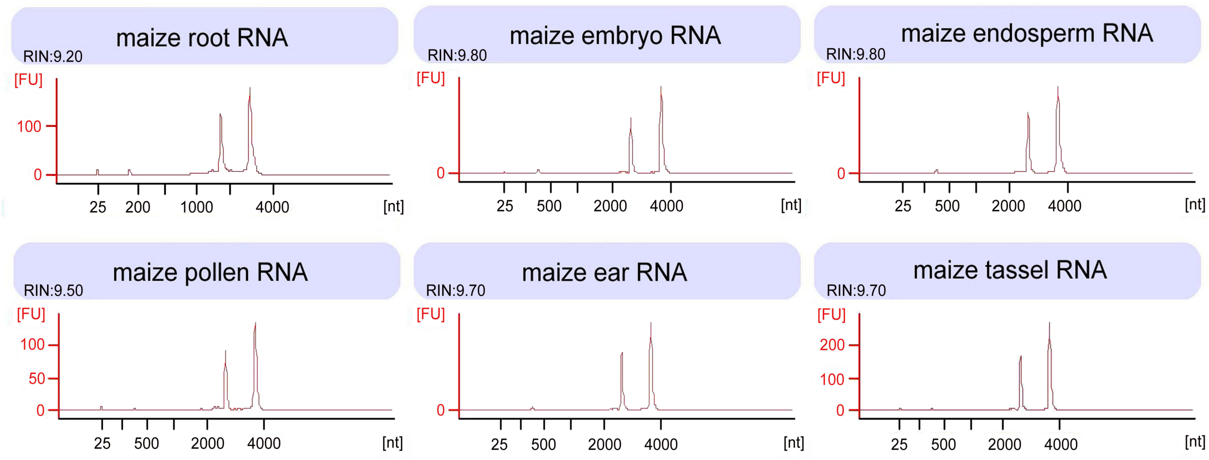

Supplementary Fig. 1 BioAnalyzer readouts of high-quality RNAs from six tissues.

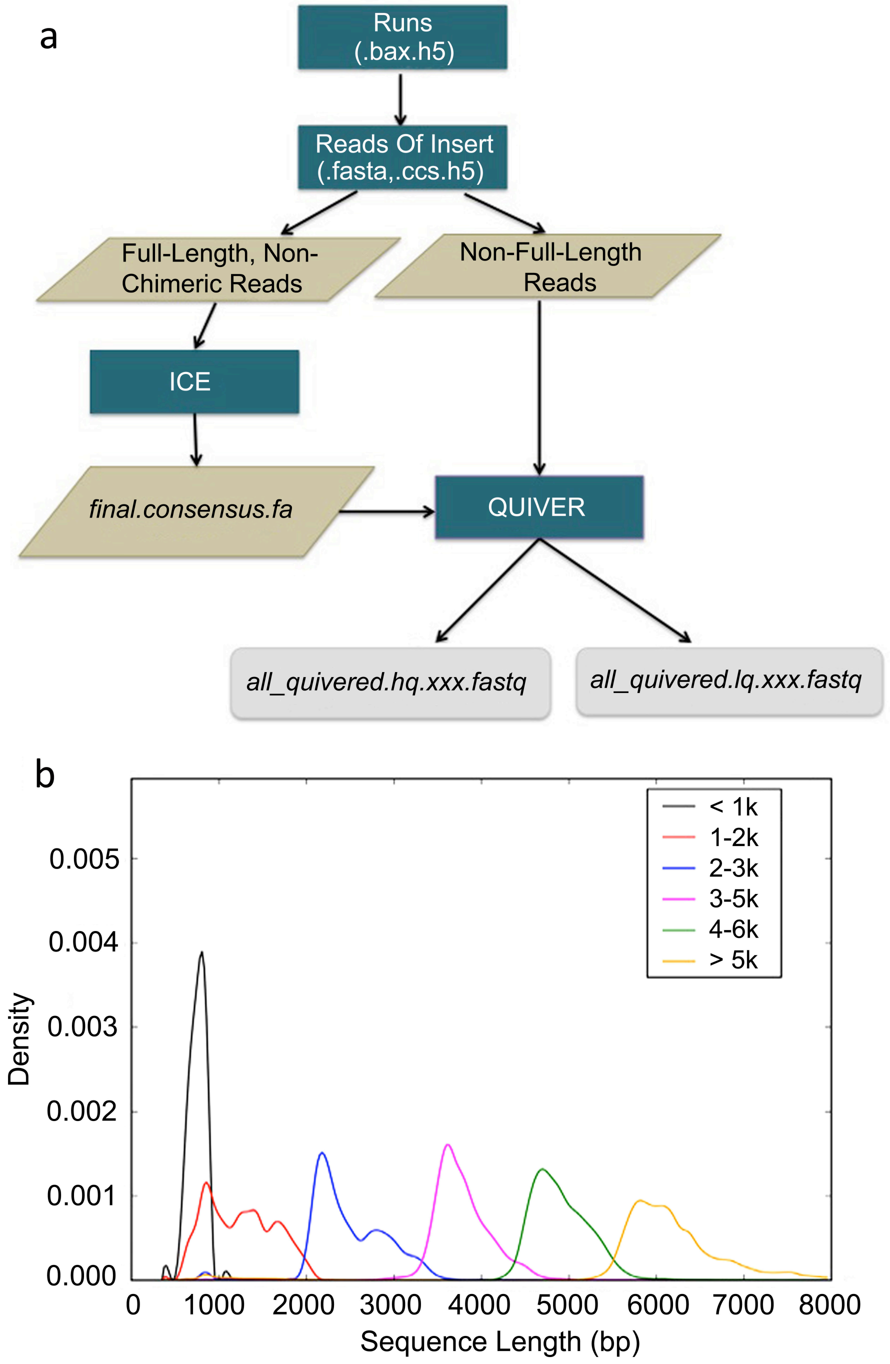

Supplementary Fig. 2 Iso-Seq workflow for data processing and size range of each library.

a) Workflow for data processing. b) Size range of each size-fractionated library.

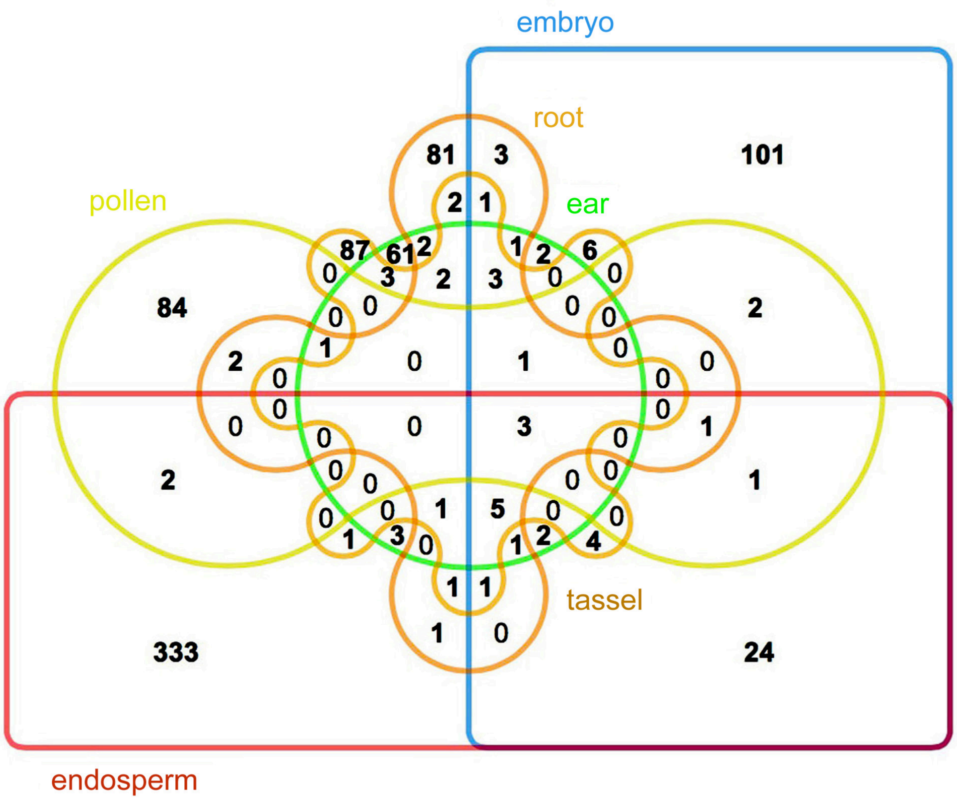

Supplementary Fig. 3 Venn diagram of TE-like PacBio isoforms in six tissues.

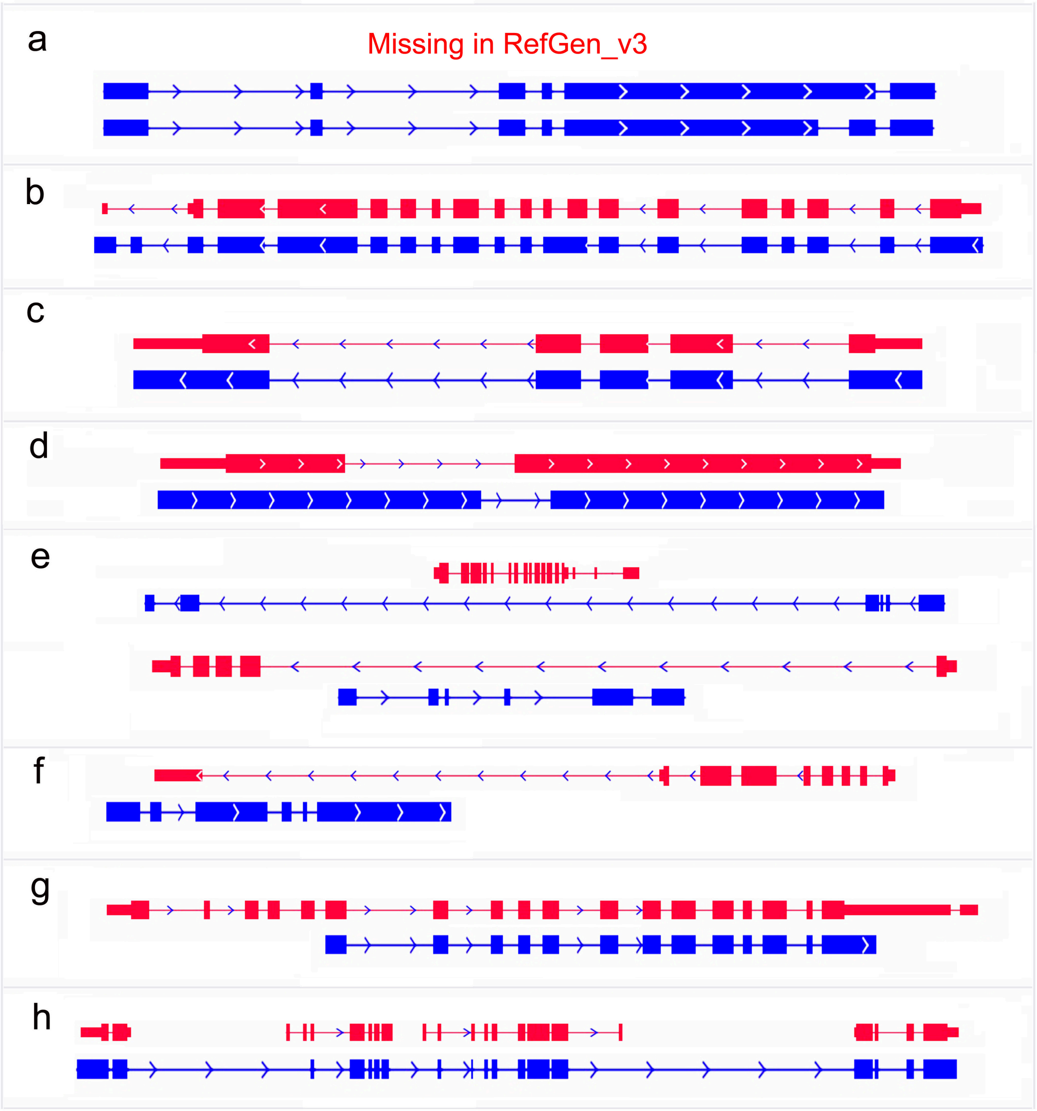

Supplementary Fig. 4 Schematic of eight groups of PacBio isoforms.

a) PacBio isoforms missing in RefGen_v3. b) Novel isoforms of known RefGen_v3 genes. c) Same isoforms as existing RefGen_v3 genes. d) Exonic overlap with RefGen_v3 genes without shared splice sites. e) RefGen_v3 isoforms located in Iso-Seq introns (upper) and Iso-Seq isoforms located in RefGen_v3 introns (lower). f) Exonic overlap with RefGen_v3 on opposite strand. g) Iso-Seq isoforms contained in RefGen_v3 isoforms. h) Falsely split RefGen_v3 gene models. * Red: RefGen_v3; Blue: PacBio Iso-Seq.

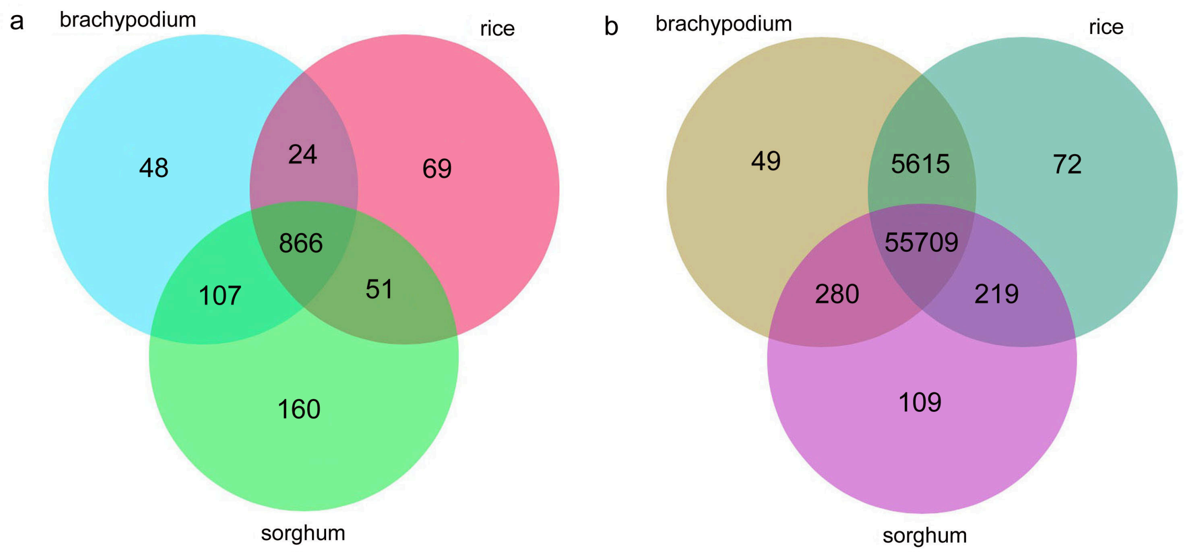

Supplementary Fig. 5 Venn diagram of protein-coding potential of novel isoforms from novel loci and novel isoforms from known genes based on comparative genomics.

a) Venn diagram of novel isoforms from novel loci. b) Venn diagram of novel isoforms from known genes.

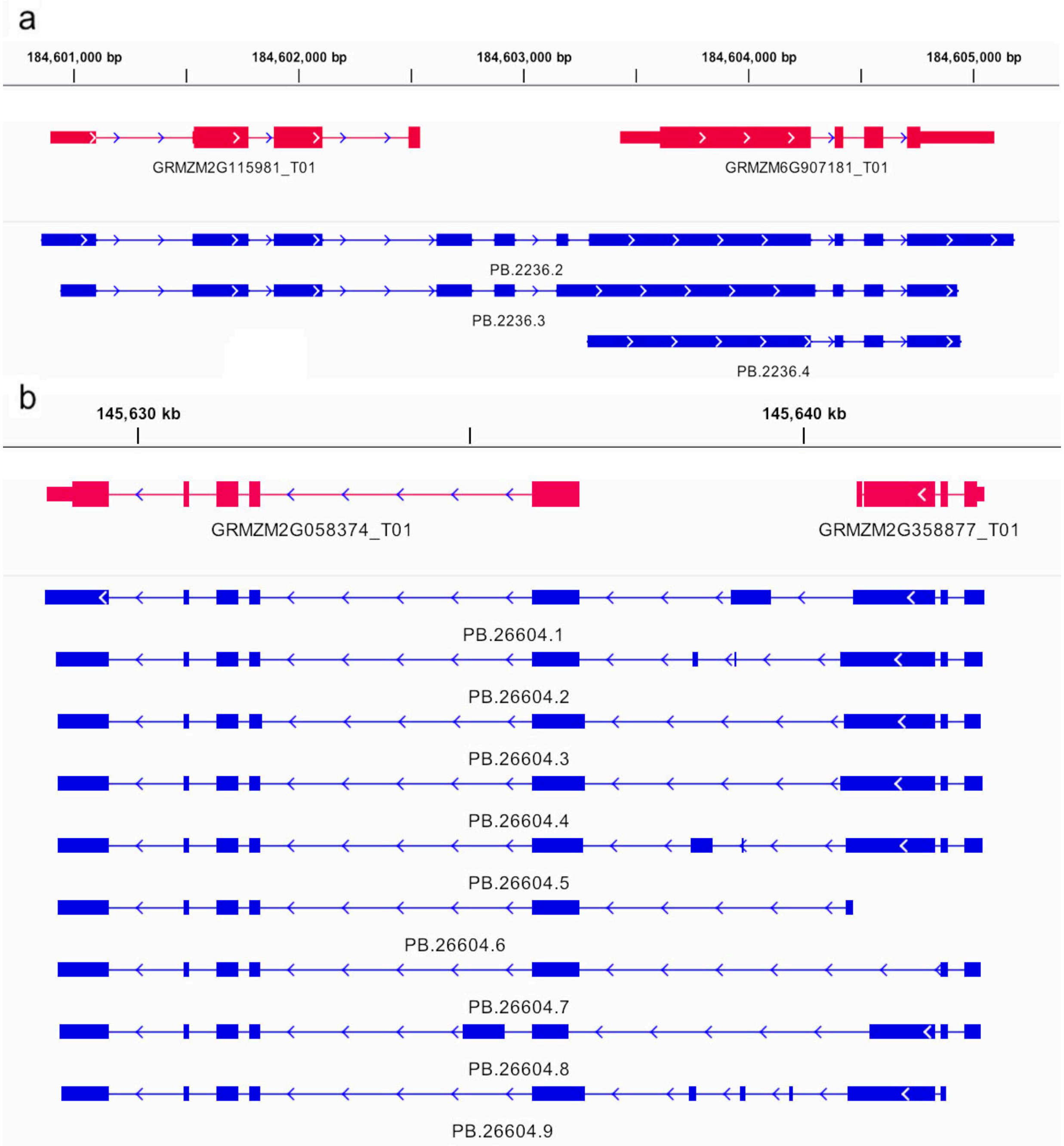

Supplementary Fig. 6 Validation of split gene models from Gramene b46 release.

a) Partially incorrect split gene models. b) Completely incorrect split gene models.

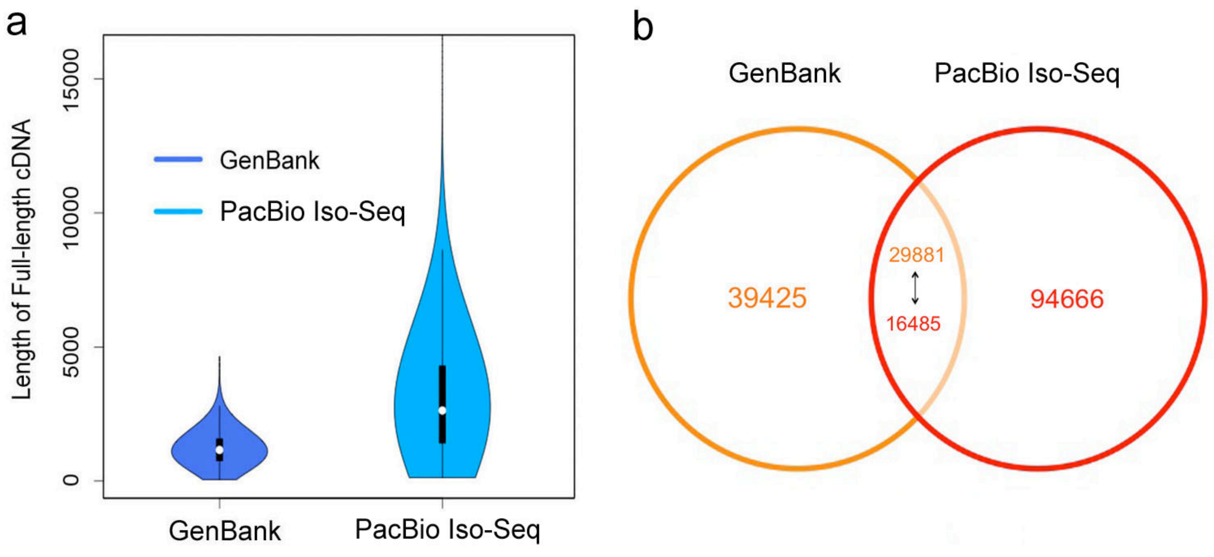

Supplementary Fig. 7 Comparison between GenBank full-length cDNAs and PacBio Iso-Seq full-length cDNAs.

a) Comparison of length distributions of GenBank and PacBio Iso-Seq full-length cDNAs. b) Overlap of GenBank and PacBio Iso-Seq full-length cDNAs.

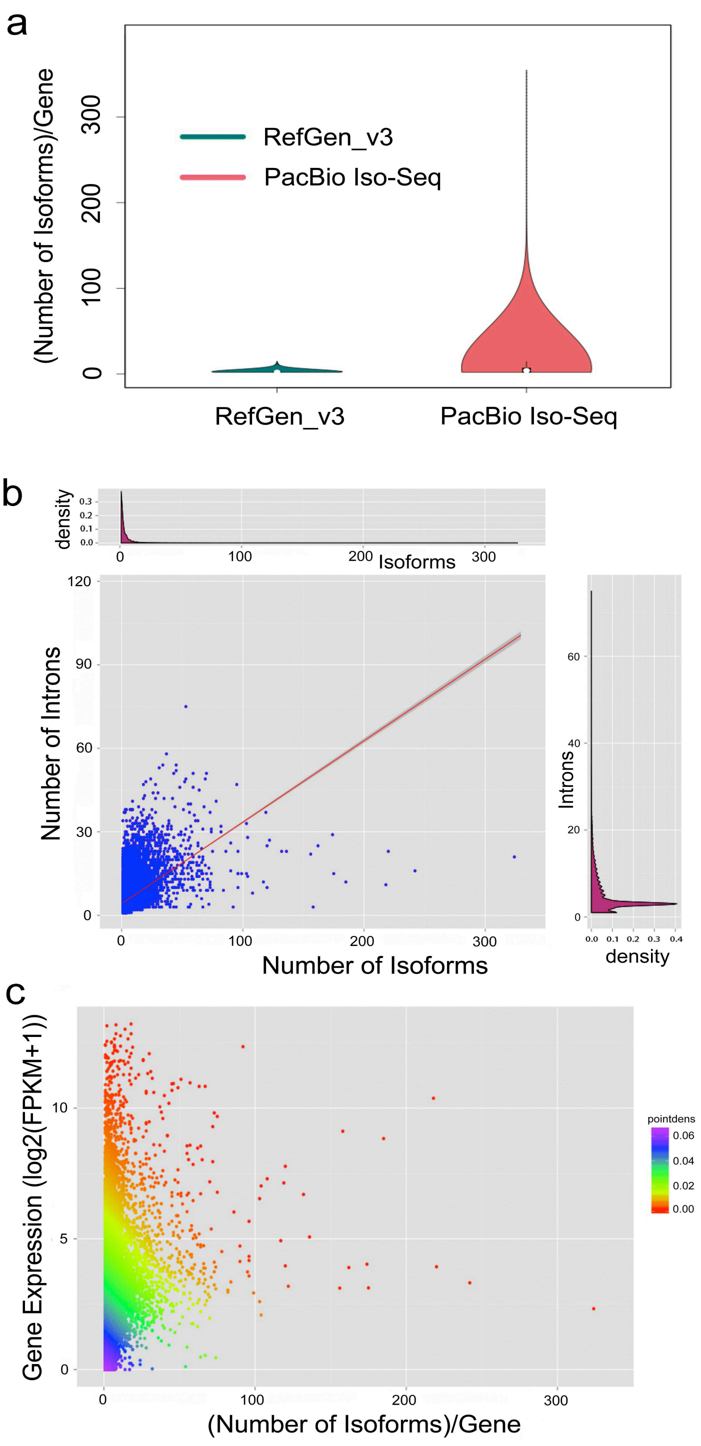

Supplementary Fig. 8 General features of PacBio isoforms.

a) Comparison of number of isoforms between RefGen_v3 and PacBio data. b) Correlation of number of introns with number of isoforms. c) Correlation of number of isoforms with gene expression value.

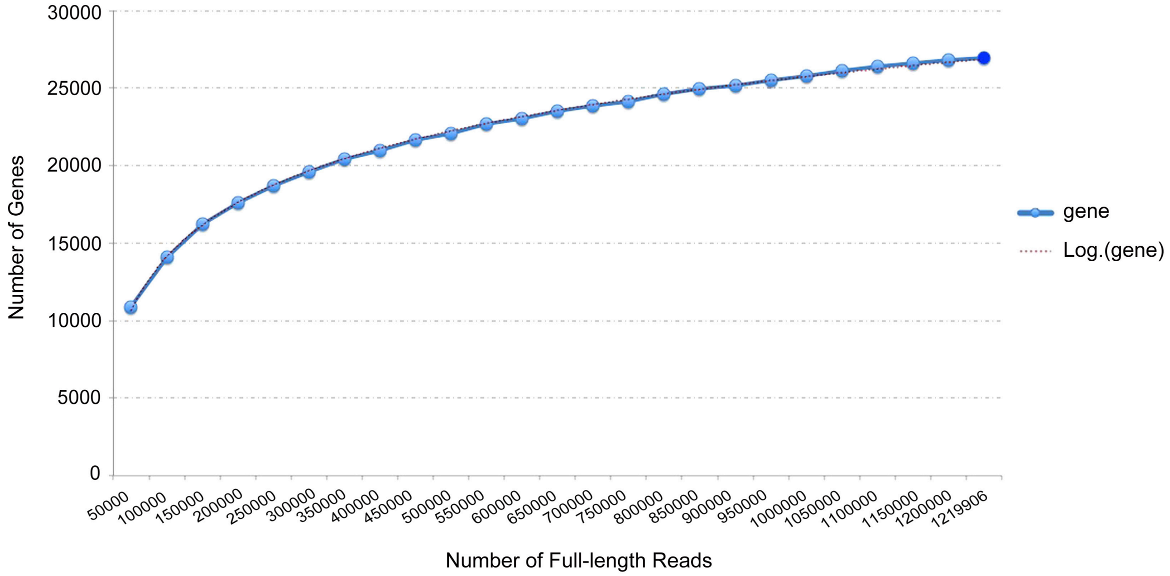

Supplementary Fig. 9 Rarefaction analysis of covered genes with number of full-length reads.

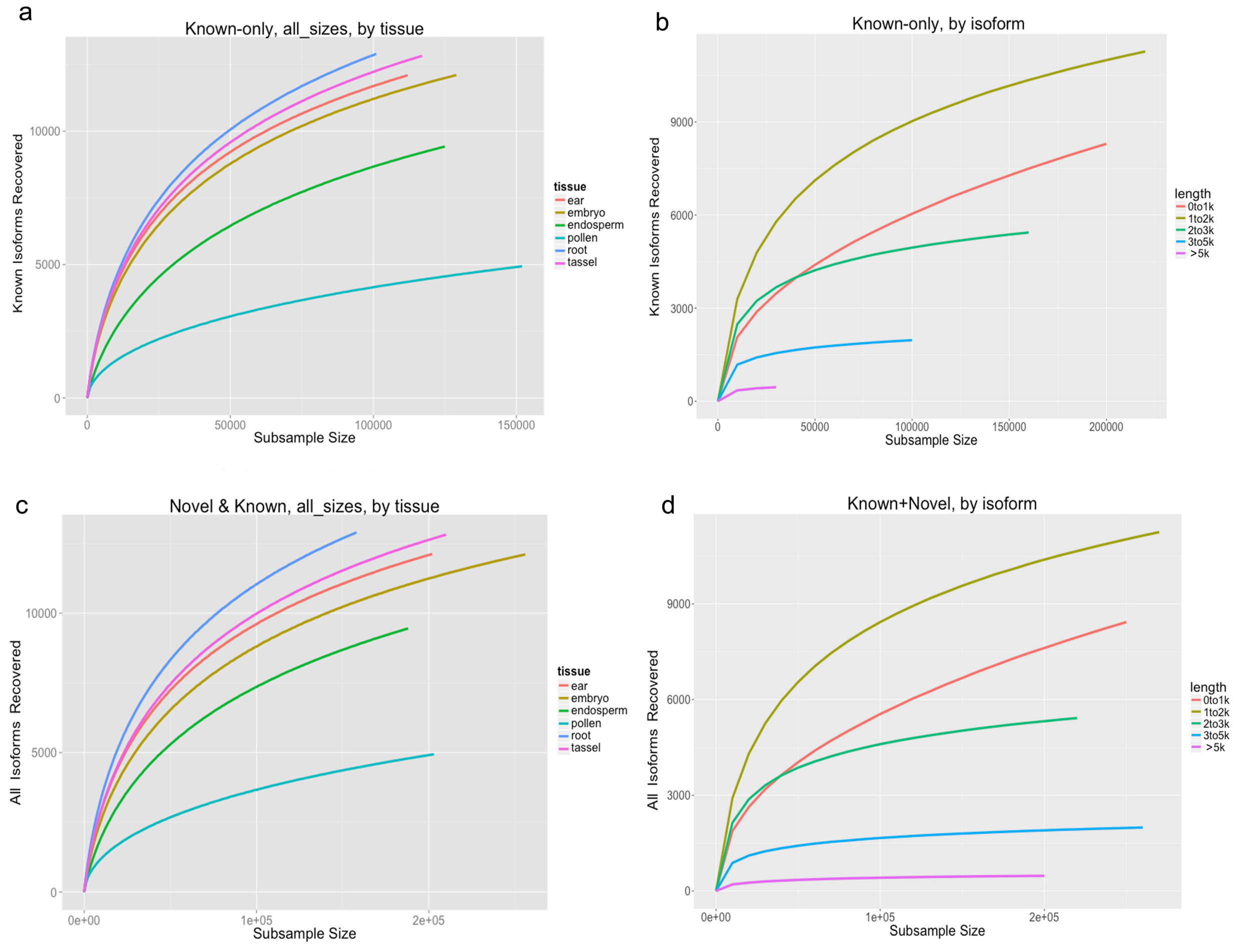

Supplementary Fig. 10 Rarefaction analysis of PacBio isoforms against annotated known RefGen_v3 isoforms.

a) Rarefaction analysis of known transcripts by each tissue. b) Rarefaction analysis of known transcripts by each size. c) Rarefaction analysis of known and novel transcripts by each tissue. d) Rarefaction analysis of known and novel transcripts by each size.

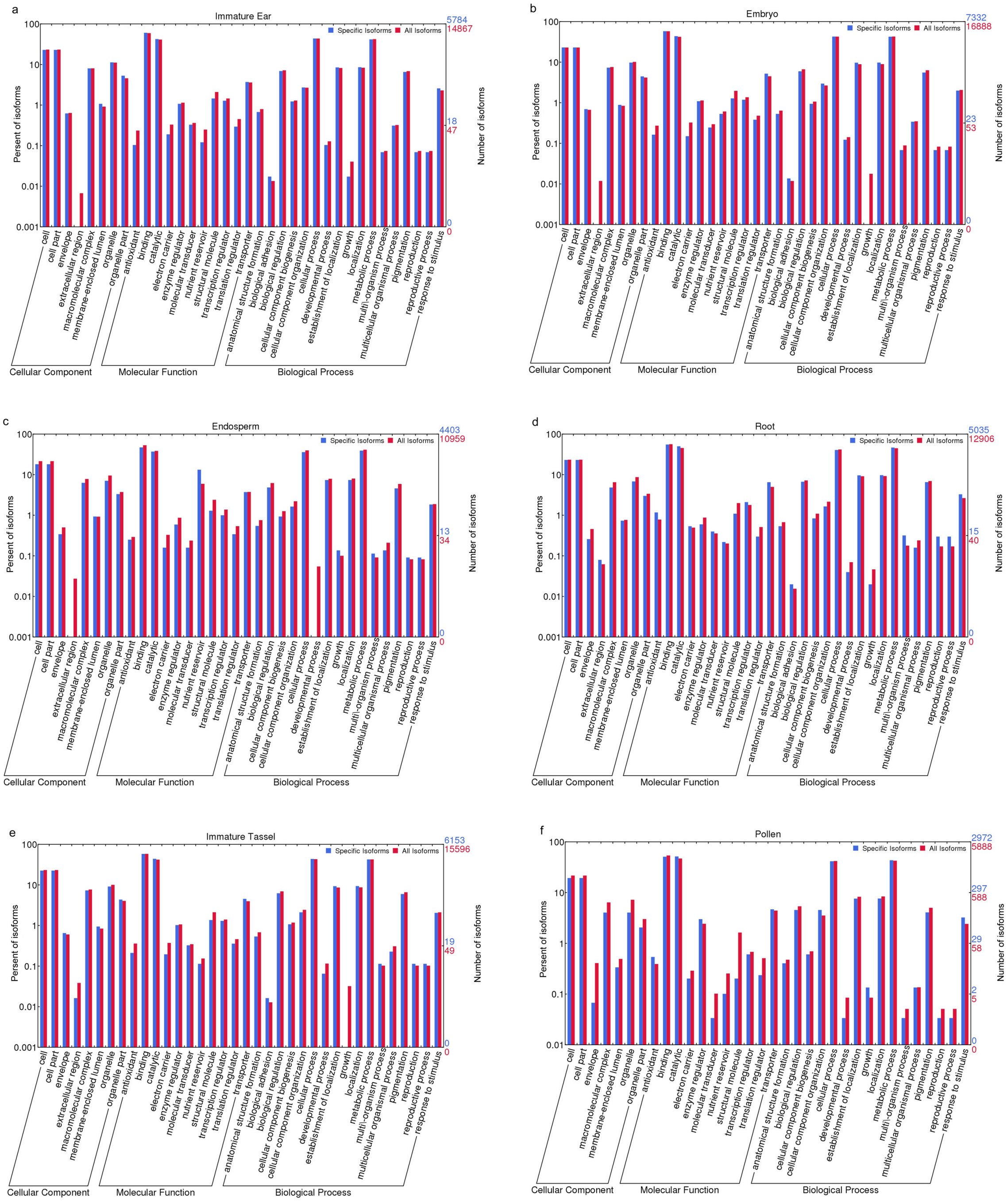

Supplementary Fig. 11 Gene ontology enrichment analysis of specific isoforms in each tissue.

a) Immature ear. b) Embryo. c) Endosperm. d) Root. e) Immature tassel. f) Pollen. The p-value is below the significant level of 0.05.

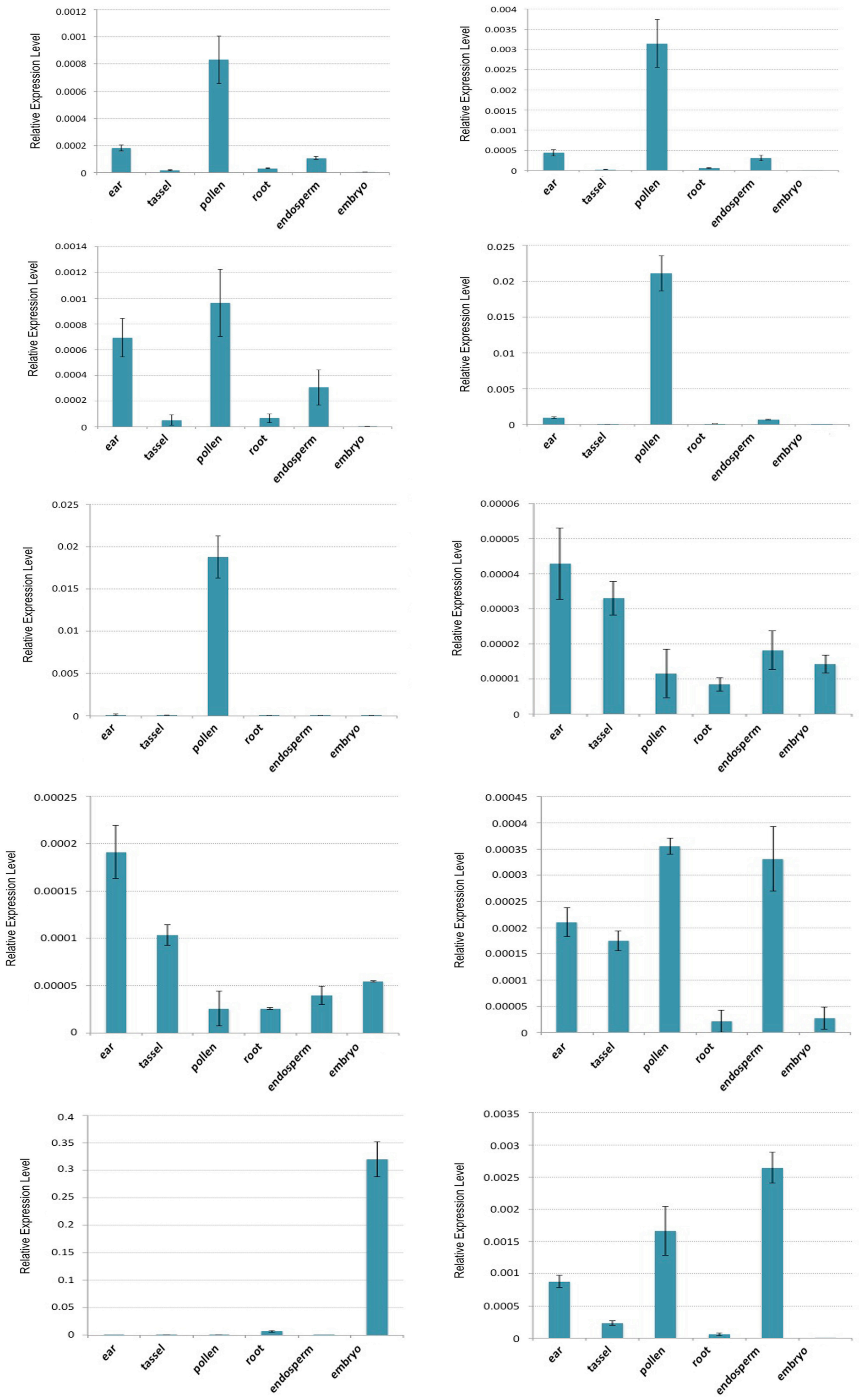

Supplementary Fig. 12 qPCR validation of 10 randomly selected intron-retention events.

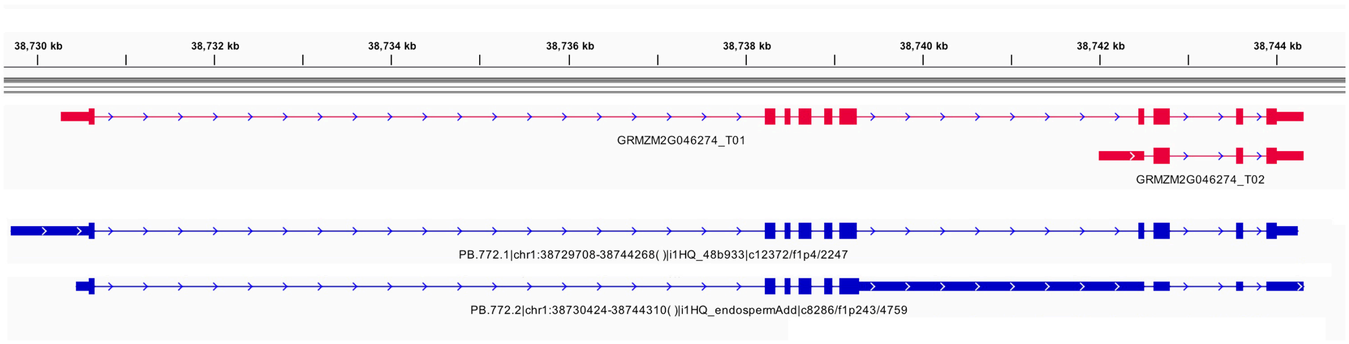

Supplementary Fig. 13 Example of ORF change caused by intron retention.

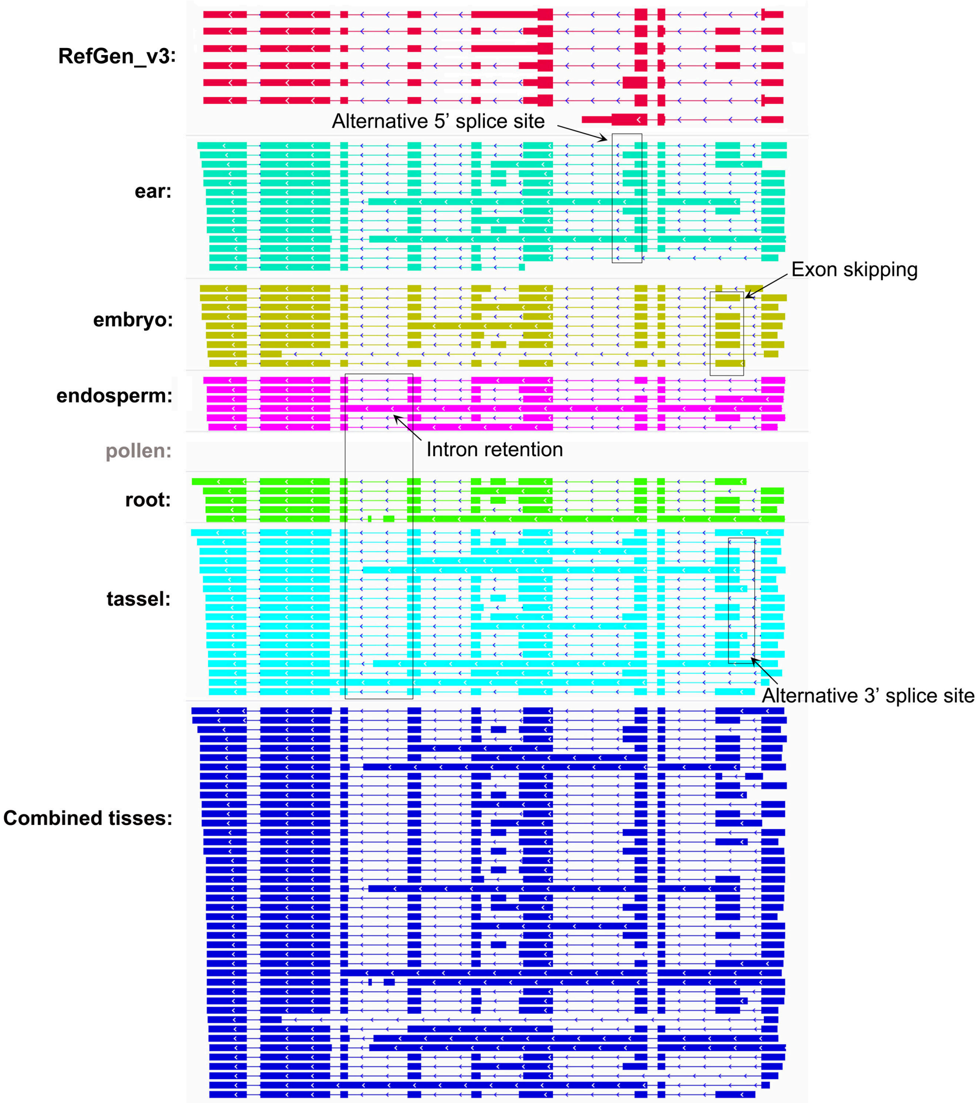

Supplementary Fig. 14 IGV visualization of different splicing modes in one gene.

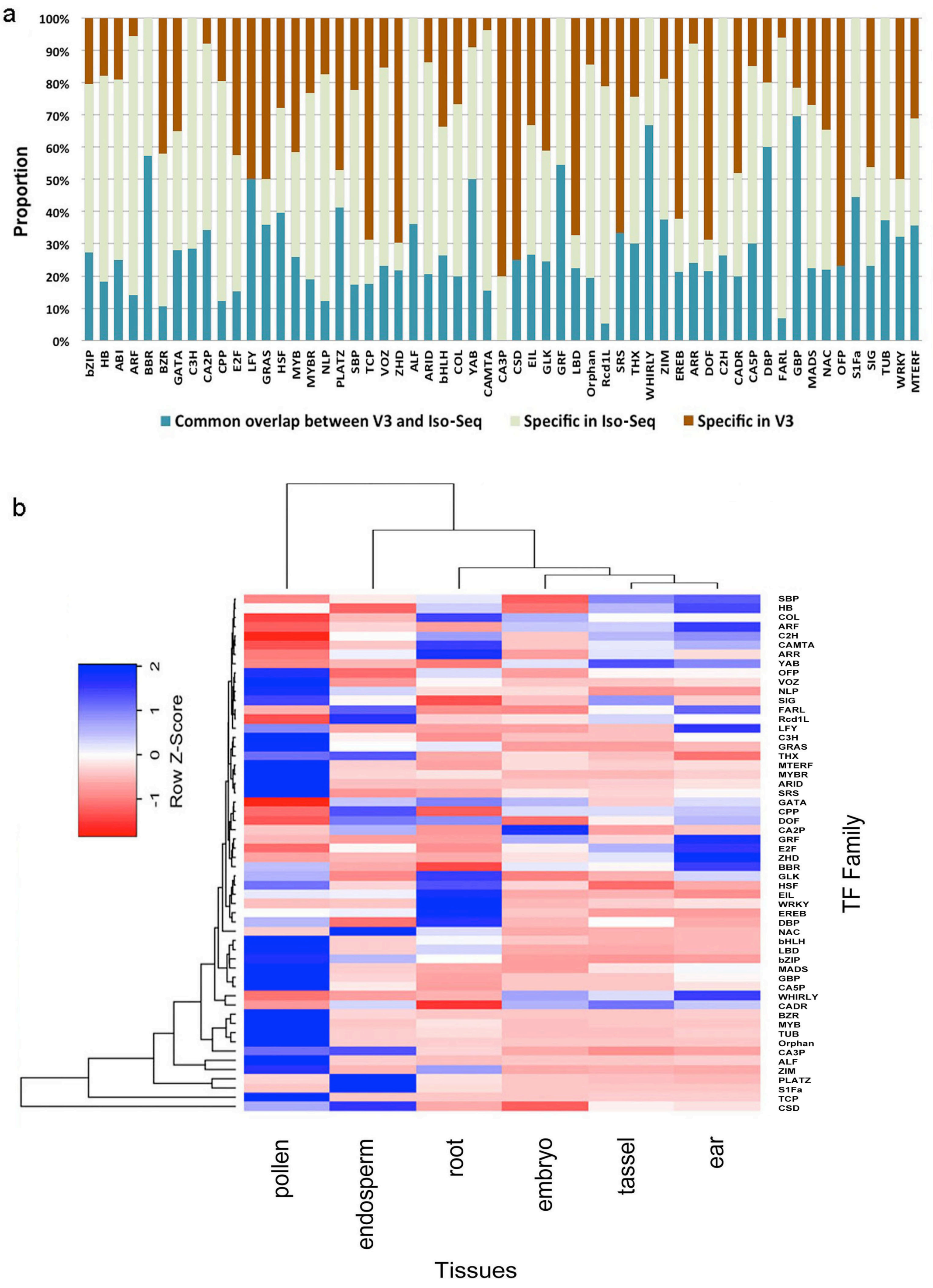

Supplementary Fig. 15 Transcription factors change from long read sequencing.

a) Comparison of known and novel isoforms identified by PacBio Iso-Seq with the RefGen_v3 annotation. b) Heatmap of expression of 57 transcription factor families.

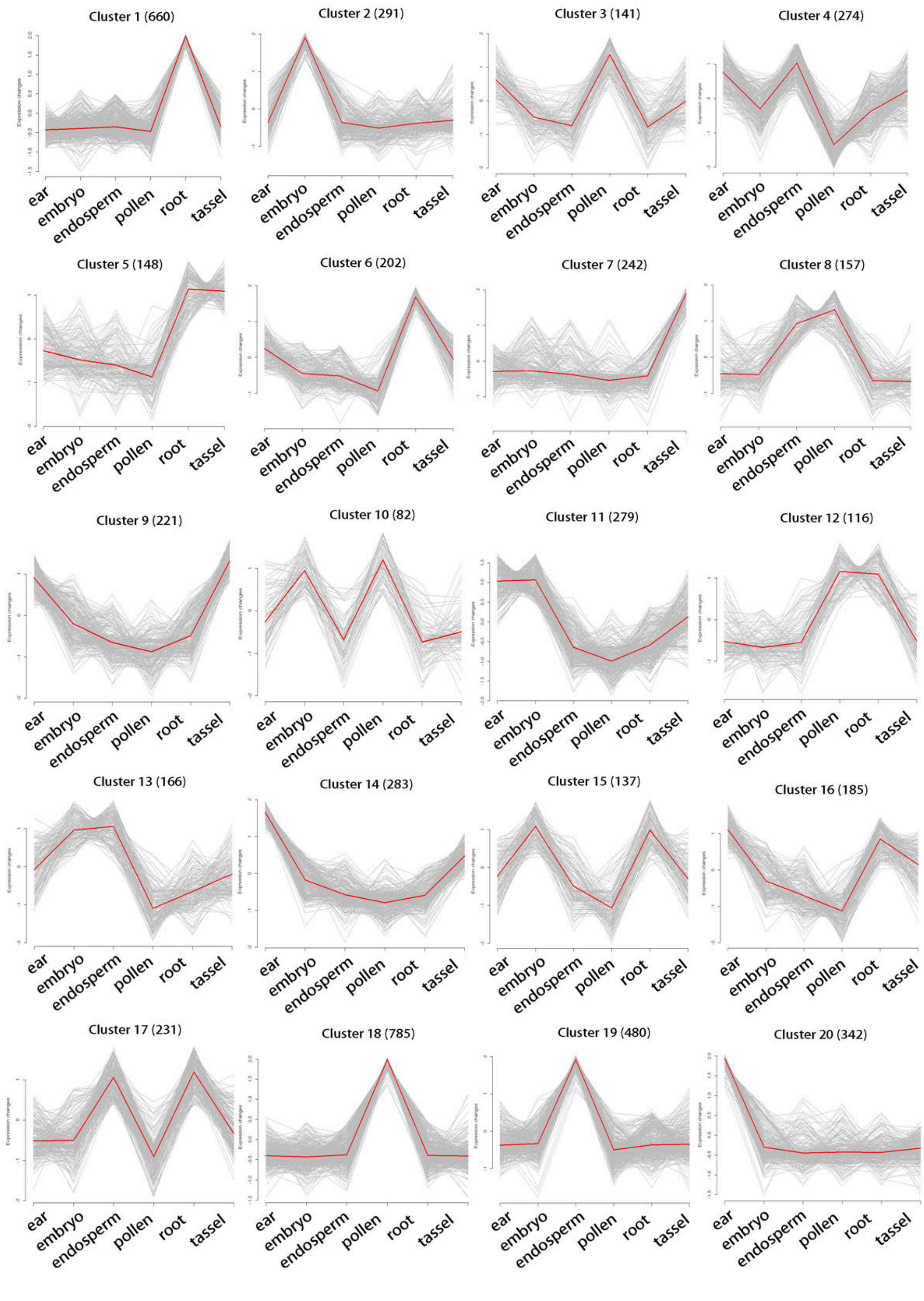

Supplementary Fig. 16 Mfuzz cluster of 5423 transcription factors identified by PacBio Iso-Seq based on their expression patterns.

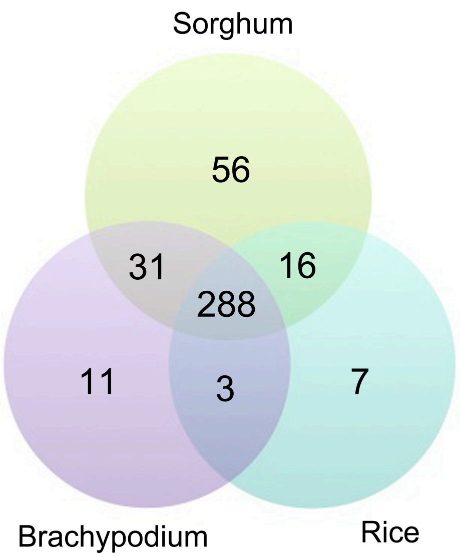

Supplementary Fig. 17 Venn diagram of discarded potential protein-coding lncRNAs.

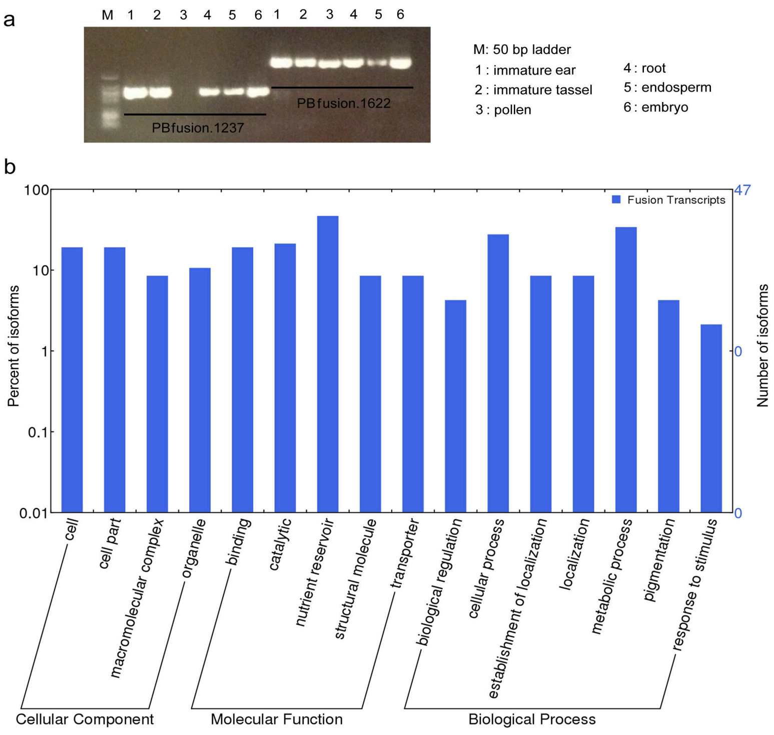

Supplementary Fig. 18 Validation and gene ontology analysis of fusion transcripts.
a) PCR validation of fusion transcripts by agarose gel electrophoresis. b) Gene ontology analysis of fusion transcripts.

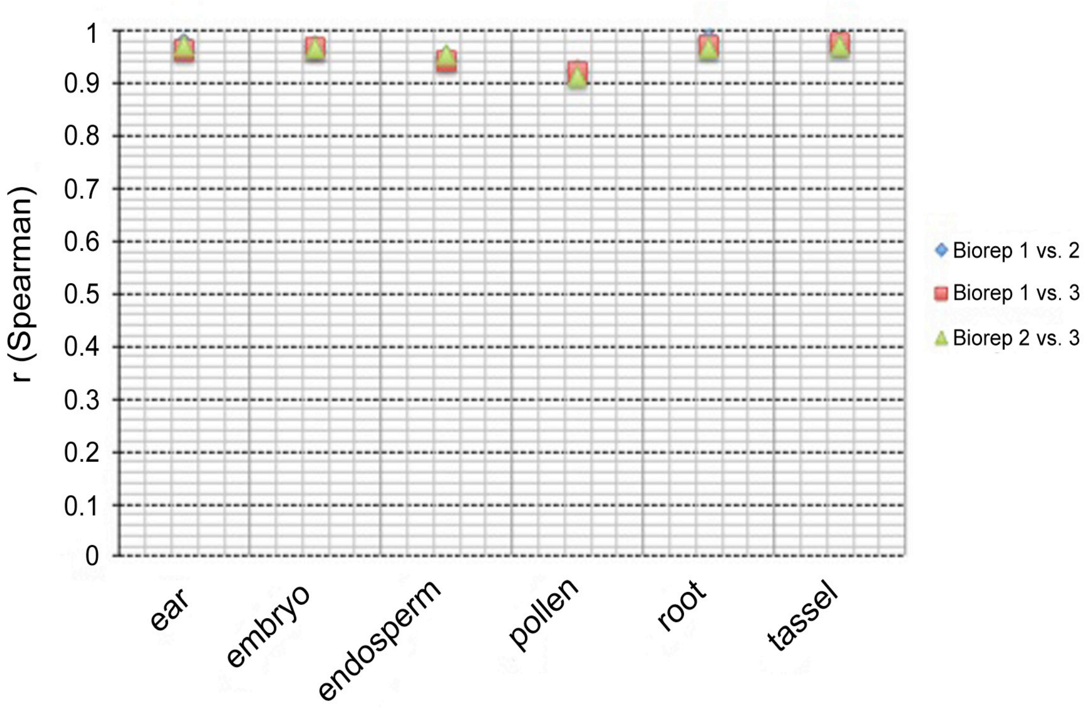

Supplementary Fig. 19 Spearman correlation of three biological replicates for each tissue.

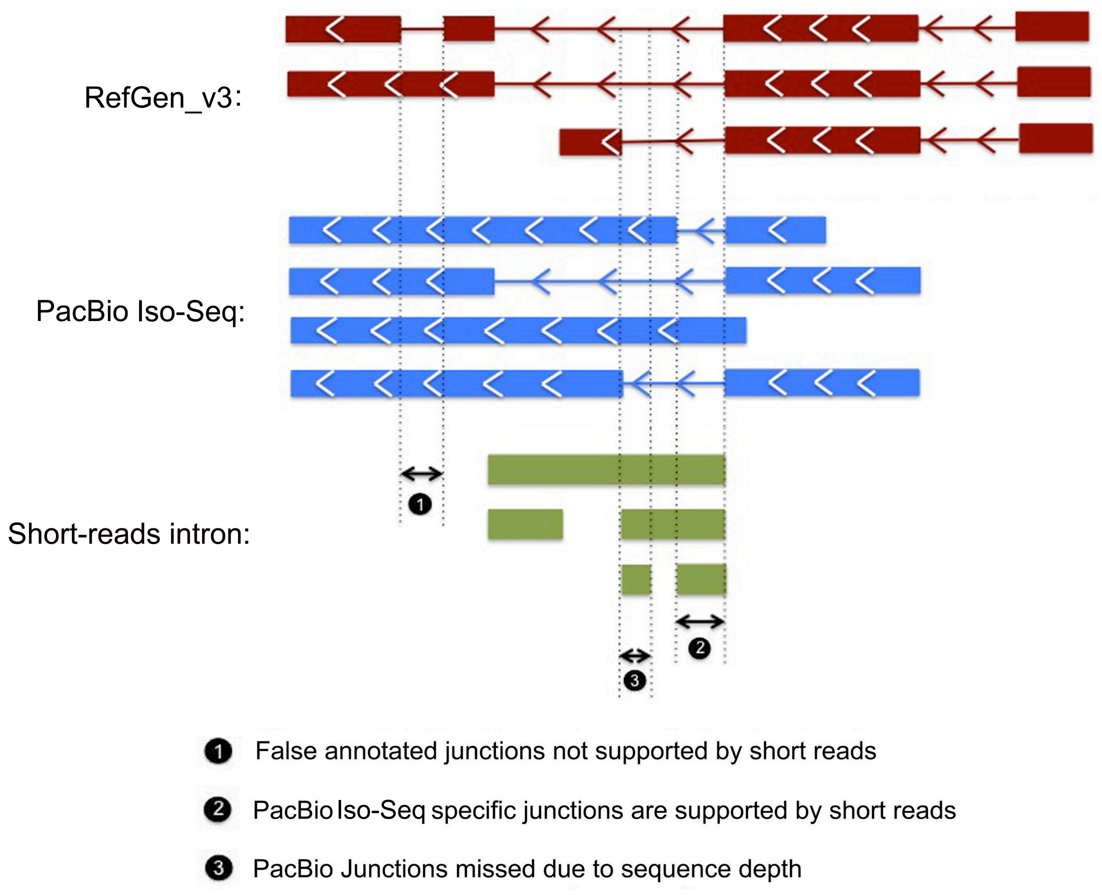

Supplementary Fig. 20 Schematic of comparison of splicing junctions between PacBio Iso-Seq and Illumina short-reads RNA-seq.

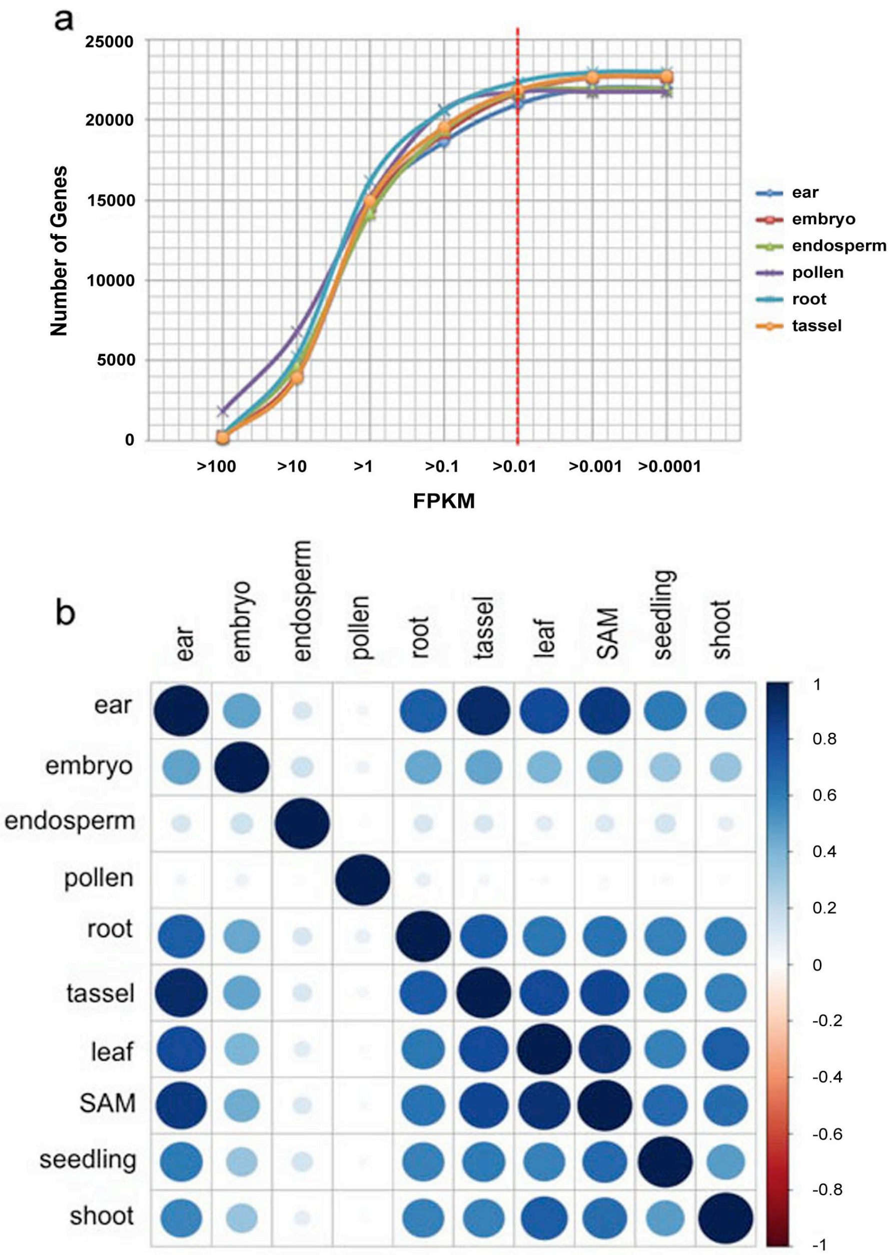

Supplementary Fig. 21 Cutoff of expression value and hierarchical cluster analysis of RNA-seq data from different tissues.

a) Cutoff of expression based on saturation analysis. b) Hierarchical cluster analysis of RNA-seq data from different tissues (leaf: SRR504466, SRR504467; shoot apical meristem (SAM): SRR504480, SRR504481; seedling: SRR1553275, SRR1553315; shoot: SRR445382).

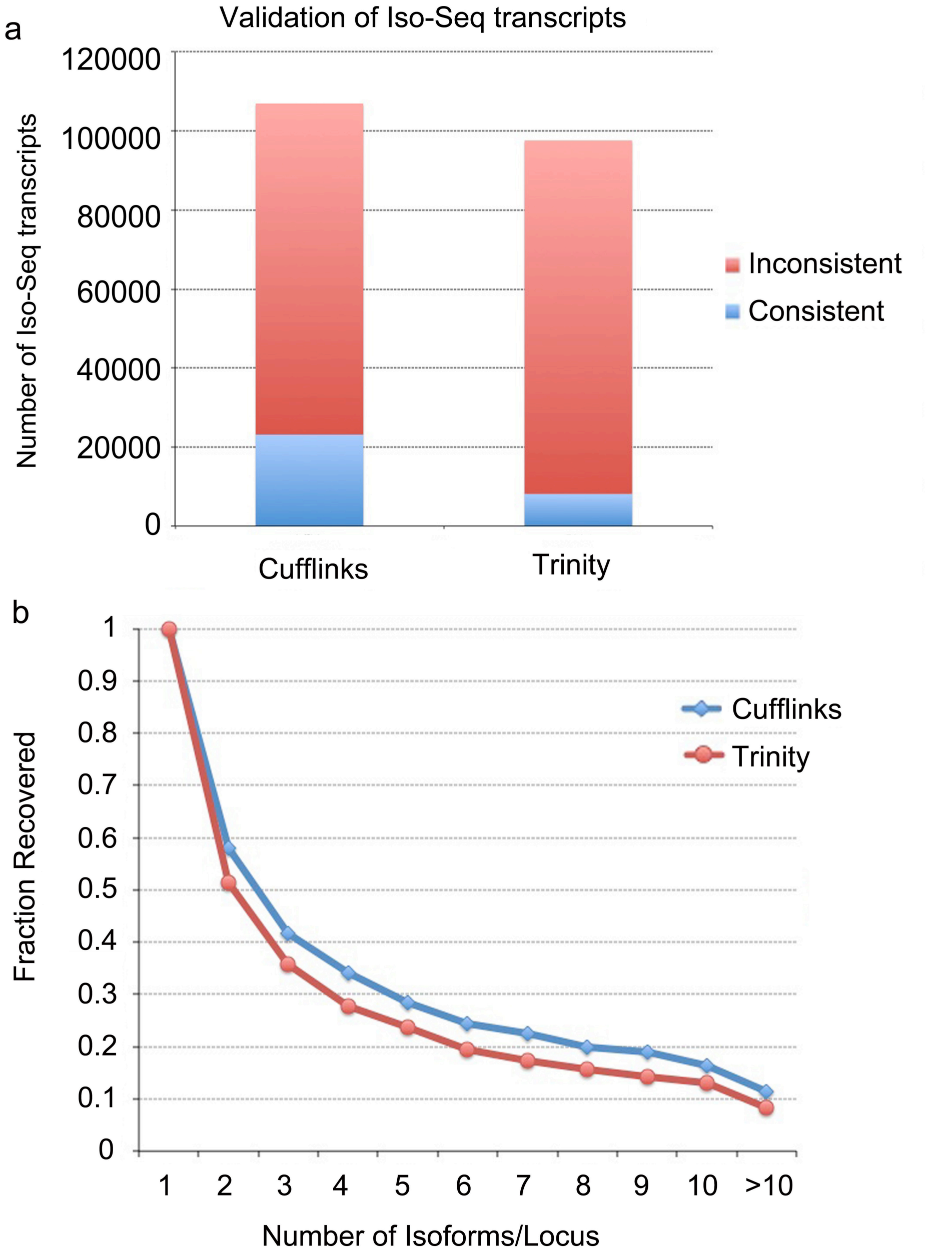

Supplementary Fig. 22 Evaluation of short-reads transcripts reconstruction against PacBio Iso-Seq transcripts.

a) Number of PacBio Iso-Seq transcripts validated by short-reads assembled transcripts. An Iso-Seq transcript is validated as an exact match of an assembled transcript if they share exactly the same number of exons and donor-acceptor sites. b) Fraction of Iso-Seq transcripts recovered (sensitivity) by each short-read assembler as a function of isoform complexity.

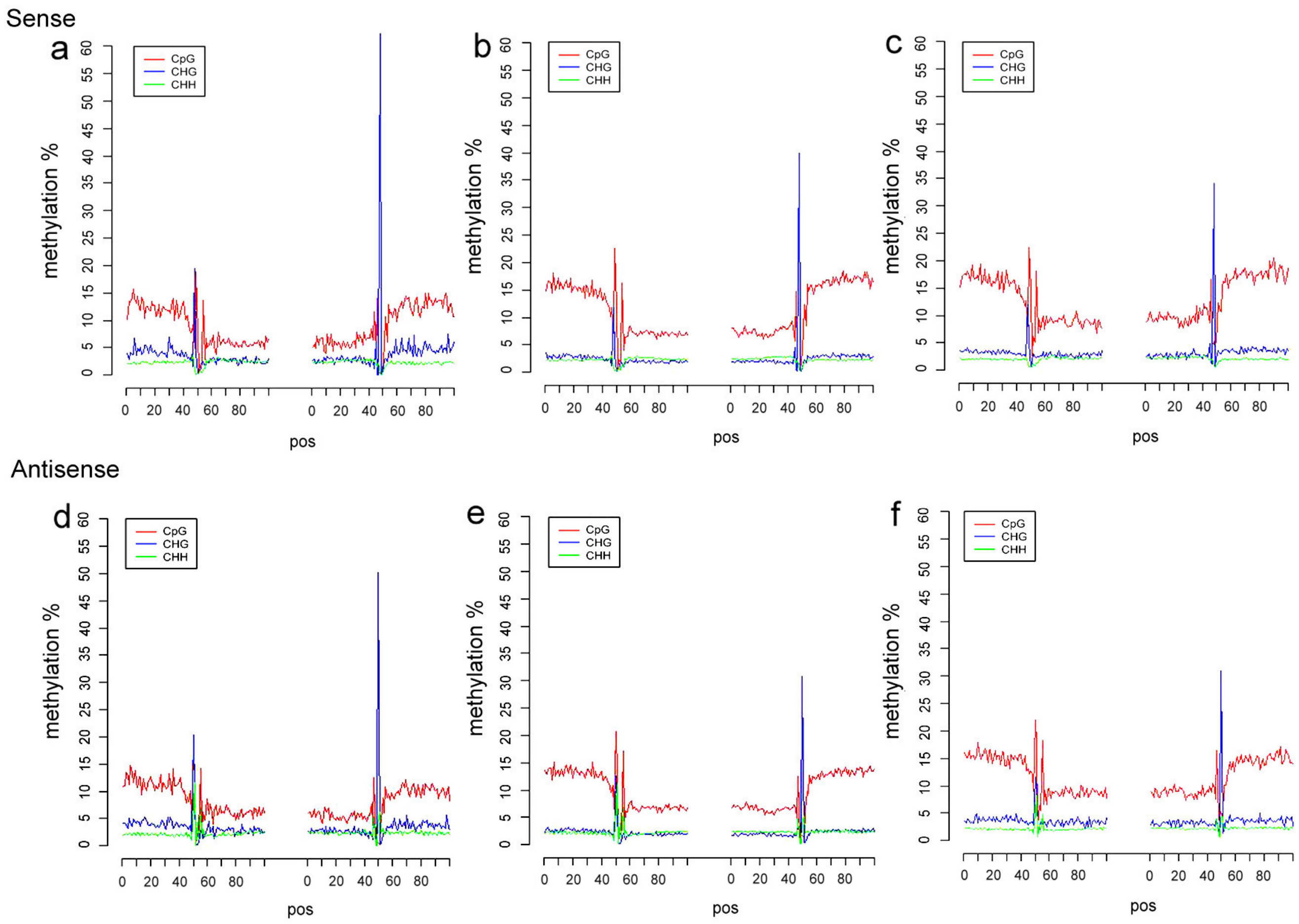

Supplementary Fig. 23 DNA methylation level at splice sites on sense and antisense strands of different isoforms.

a) DNA methylation level on the sense strand at splice sites of isoforms of genes with only one isoform. b) DNA methylation level on the sense strand at splice sites of isoforms of genes with two to ten isoforms. c) DNA methylation level on the sense strand at splice sites of isoforms of genes with more than twenty isoforms. d) DNA methylation level on the antisense strand at splice sites of isoforms of genes with only one isoform. e) DNA methylation level on the antisense strand at splice sites of isoforms of genes with two to ten isoforms. f) DNA methylation level on the antisense strand at splice sites of isoforms of genes with more than twenty isoforms.

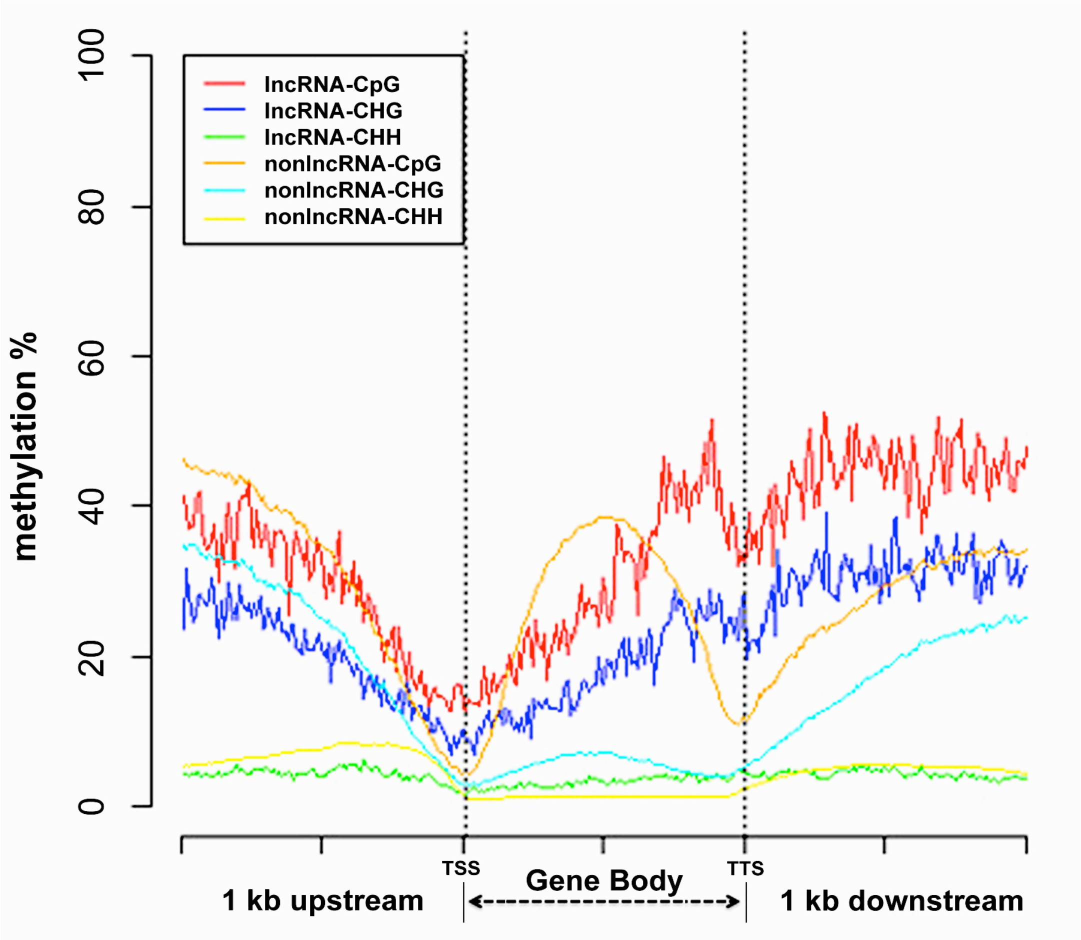

Supplementary Fig. 24 Comparison of DNA methylation level on lncRNAs and non-lncRNAs.

Supplementary Table 1. Sequence summary of PacBio SMRT Cells.

Sample	Cells	Total Reads[a]	Total FL Reads	FL length range[b]
< 1kb	7	498,344	317,017 (64%)	571-877
1–2 kb	8	664,767	373,824 (56%)	688-1918
2–3 kb	8	725,197	320,490 (44%)	2,007-3,318
3–5 kb	8	619,908	206,843 (33%)	3,359-4,498
4–6 kb	7	489,474	145,933 (30%)	4,467-5,559
>5 kb	9	718,914	189,585 (26%)	5,161-7,771
Total	47	3,716,604	1,553,692	645-6,260

a: Reads Of Insert Reads. b: length range from 5% to 95%.

FL = barcode + cDNA primer seen on both 5' and 3' end. For 3' end, also required to see polyA tail before primer.

Supplementary Table 2. 16-mer barcode sequences used for PacBio barcoding libraries.

Oligo	Sequence
dT_BC1	AAGCAGTGGTATCAACGCAGAGTAC**tcagacgatgcgtcat**TTTTTTTTTTTTTTTTTTTTTTTTTTTTTTVN
dT_BC2	AAGCAGTGGTATCAACGCAGAGTAC**ctatacatgactctgc**TTTTTTTTTTTTTTTTTTTTTTTTTTTTTTVN
dT_BC3	AAGCAGTGGTATCAACGCAGAGTAC**tactagagtagcactc**TTTTTTTTTTTTTTTTTTTTTTTTTTTTTTVN
dT_BC4	AAGCAGTGGTATCAACGCAGAGTAC**tgtgtatcagtacatg**TTTTTTTTTTTTTTTTTTTTTTTTTTTTTTVN
dT_BC5	AAGCAGTGGTATCAACGCAGAGTAC**gatctctactatatgc**TTTTTTTTTTTTTTTTTTTTTTTTTTTTTTVN
dT_BC6	AAGCAGTGGTATCAACGCAGAGTAC**acagtctatactgctg**TTTTTTTTTTTTTTTTTTTTTTTTTTTTTTVN

Supplementary Table 3. Primers designed for qPCR validation.

geneID	locus	Position of IR	PCR region	Left primer (5'-3')	Right primer (5'-3')
PB.67.12	chr1:2,993,230-3,023,853	3rd exon	chr1:3005450-3006465	GTGGGATTTGGGGTAGGAAT	CACATGCTCTGCAATTTTGA
PB.124.2	chr1:4,707,916-4,715,571	8th exon	chr1:4711238-4712276	AGAAAACGGAGGAGCAGACA	GTGAGGCTGCTGATGTTCAA
PB.277.5	chr1:10,373,464-10,404,087	2nd exon	chr1:10380645-10382275	CCAGGATGACCCAAGATGAT	AACCGTGGCTACAAATCAGG
PB.312.40	chr1:12,973,797-12,981,452	3rd/9th exon	chr1:12975953-12976581	TATGGCAACGTTTGCTCAAC	CGGGCGATAAATATTCGAGA
PB.491.2	chr1:22,732,999-22,740,654	2nd exon	chr1:22736101-22736320	TGAGTTAAATCTAGGCTATG	CCTGCCAAAAAATATGATTG
PB.495.3	chr1:22,883,020-22,898,331	1st exon	chr1:22887938-22889135	TTTCTCGGGAGATTGTACGG	CGAGTGTCAACAGCAGCAAT
PB.6082.25-1	chr2:3,612,437-3,624,519	1st exon	chr2:3614569-3616720	CGCTGGCTTGCTCCTTATAC	GTTGGCATGTCCTTCTGGTT
PB.6082.25-2	chr2:3,612,437-3,624,519	3rd exon	chr2:3617562-3619120	GGTTGACCTTTCGGGTATGA	TCAATCAAGATGGCCAACAA
PB.6118.4	chr2:4,581,481-4,593,563	1st exon	chr2:4585821-4586164	CACGGATTTGGAGGTGAGTT	TCTACGACAACGACCACAGG
PB.6126.24	chr2:4,818,878-4,843,044	3rd exon	chr2:4826939-4829291	TTCAAACGGTGTTTGGTTCA	GGGTGAAGCAGAGCAGTAGC

Supplementary Table 4. Primers designed for PCR and Sanger sequencing validation of candidate fusion transcripts.

fusionID	Left primer (5'-3')	Right primer (5'-3')
PBfusion.1095	GGGCCTACCATGGTGGTGAC	GGCAAAGCACAGGTAACTCA
PBfusion.1237	CCTGGTGTTGGAGATCACCA	CCCAAAGATCTTGGCAATAAC
PBfusion.1310	ATGGTAATTCGAACTGTGAA	ACCATAAGAGCAGAGCGGAA
PBfusion.1520	ATGGAGTATAGACTGAGTTT	AAACGGCCTACAAAGCAGCTC
PBfusion.1580	ATAACTTGACGGATCGCACGG	CATAGAAACATATAGCTGGG
PBfusion.1584	GCGCACGACGGGCTCATGGT	ATTGACACACACATGTACAATA
PBfusion.1622	ATGGTGACCCAAAATCAGTT	CAGCAGGGTTATGAACTCCAC
